# The Colours of Octopus: Using Spectral Data to Measure Octopus Camouflage

**DOI:** 10.3390/vision6040059

**Published:** 2022-09-22

**Authors:** Luis Nahmad-Rohen, Yusuf H. Qureshi, Misha Vorobyev

**Affiliations:** 1 Leigh Marine Laboratory, Institute of Marine Science, University of Auckland, Leigh, Auckland 0985, New Zealand; 2 Optometry and Vision Science, Faculty of Medical and Health Sciences, University of Auckland, Grafton, Auckland 1023, New Zealand

**Keywords:** octopus, camouflage, spectrometry, colour matching, visual systems

## Abstract

No animal can so effectively camouflage in such a wide range of environments as the octopus. Thanks to their highly malleable skin, they are capable of adapting their body patterns to the brightness and texture of their immediate environment, and they often seemingly match the colour of background objects. However, octopuses are colour-blind as their eyes have only one type of visual pigment. Therefore, chromatophores in their skin are likely to respond to changes in brightness, not chromaticity. To determine whether octopuses actually match background colours, we used a SpectraScan^®^ PR-655 spectroradiometer to measure the reflectance spectra of *Octopus tetricus* skin in captivity. The spectra were compared with those of green algae, brown algae, and sponges—all of these being colourful objects commonly found in the octopus’s natural environment. Even though we show that octopuses change both lightness and chromaticity, allowing them to potentially camouflage in a wide range of backgrounds in an effective manner, the overall octopus colours did not reach the same level of saturation compared to some background objects. Spectra were then modelled under the visual systems of four potential octopus predators: one dichromatic fish (Heller’s barracuda), two trichromatic fish (blue-spotted stingray and two-spotted red snapper), and one tetrachromatic bird (wedge-tailed shearwater). We show that octopuses are able to match certain background colours for some visual systems. How a colour-blind animal is capable of colour-matching is still unknown.

## 1. Introduction

Octopuses have exceptionally refined abilities to camouflage on any background, matching texture and colour of backgrounds in a fraction of a second [1,2,3,4,5]. However, octopus and other cephalopods—with the possible exception of some mesopelagic squids [6,7,8,9]—have only one type of visual pigment, and behavioural experiments have demonstrated that octopus and cuttlefish are colour blind [10,11]. Therefore, the ability of octopuses to camouflage against coloured backgrounds is difficult to explain. Here we investigate a range of reflectance spectra of octopus (*Octopus tetricus*), compare them with the spectra of differently coloured backgrounds, and model the colours of octopus and backgrounds as they are seen by potential predators, while reflectance spectra of cuttlefish have been measured [12,13,14], the reflectance spectra of octopus have not been previously published and the ability of octopus to achieve colour resemblance has not been demonstrated using quantitative methods.

One explanation for the remarkable ability of colour blind octopuses to resemble colour is that their skin reflects light that is, in turn, reflected from the background, allowing the octopuses to acquire the same colour as the surrounding objects (such as sand, rocks, coral, or algae) [15,16,17]. This may provide a passive form of colour camouflage. However, the passive camouflage hypothesis probably does not explain the colour resemblance of octopus because light reflected from a surface has a much lower intensity than the incident light. Therefore, any light reflected from a substrate or background object is likely to be of insufficient intensity to change the colouration of the octopus. Moreover, octopuses often imitate colours (and other features) of distant objects [18].

Cephalopods change their body colours thanks to neurally controlled elements in their skin [1,19]: iridophores that reflect light in a spectrally selective manner, chromatophores that absorb light, and leucophores that reflect light uniformly. Interactions between chromatophores, iridophores and leucophores explain variations of octopus colours [15,20]. Spectral measurements of squid skin components have shown that chromatophores and iridophores in combination produce colours which cannot be achieved by either of these structures on their own [5]. Thus, subtle changes in the iridophores’ reflected wavelength and their interaction with chromatophores may allow squid to reflect a wider range of colours. As octopus and cuttlefish skin is even more densely packed with chromatophores and iridophores than the skin of a squid, these principles could also apply to them [5]. Indeed, spectrometry has shown that spectra of cuttlefish chromatophores are similar to those of some natural substrates [5] and multispectral imaging has demonstrated that cuttlefish can achieve high-fidelity colour-match of gravel and sand for both dichromatic and trichromatic predators [13]. However, many substrates have more spectral variation than the cephalopod skin [12].

Our study species, *Octopus tetricus*, occurs in both rocky reef and soft sediment habitats in temperate waters of south-east Australia and northern New Zealand [21]. For this study we used colourful objects naturally occurring in the octopus’s environment: green algae, brown algae, and sponges. These were collected from the same location as the octopuses. To compare colours of octopus with these backgrounds we use two approaches. (i) Analysis of reflectance spectra of octopus and backgrounds. If the reflectance spectra of octopus and backgrounds were similar, then we could conclude that camouflage was successful for any visual system. (ii) Modelling of colours of octopus and backgrounds as they are seen through the eyes of potential predators. Colours can be similar for the predator eyes even when spectra differ substantially. Moreover, object colours can be discriminated by one predator but not by another given their differing colour vision systems.

Four potential octopus predators were chosen based on their shared habitat with our study species, their diet composition, and availability of data on their spectral sensitivities: Heller’s barracuda (*Sphyraena helleri*), a dichromatic pelagic fish common in Queensland and New South Wales, Australia [22] which, like other barracudas, is an opportunistic predator of fish, cephalopods, and crustaceans [22,23]; Blue-spotted stingray (*Neotrygon kuhlii*), a trichromatic shallow benthic species present in the northern coast of New South Wales, Australia [24] and Tasman Sea and western coast of North Island in New Zealand [25,26], which feeds on a wide variety of marine organisms such as molluscs and crustaceans [25,27]; Two-spotted red snapper (*Lutjanus bohar*), a large trichromatic pelagic fish present in certain areas of New South Wales (Sydney and north of Coffs Harbour) and Lord Howe Island [28,29,30], which preys on fish, crustaceans, and cephalopods [28]; and Wedge-tailed shearwater (*Puffinus pacificus*), a tetrachromatic diving bird occurring in the western coast of Australia, the Tasman Sea, and the New Zealand region [31,32], which can dive to depths of more than 60m to forage for fish and cephalopods [33].

To analyse reflectance spectra, a principal component analysis (PCA) was used. A PCA reduces the dimensionality of spectra and allows us to plot spectra as points in low-dimensional spaces where the principal components (PCs) are plotted along the coordinate axes. Generally, different sets of spectra belong to different spaces. Furthermore, for a set of spectra, a PCA gives an optimal method of approximating spectra as a weighted sum of principal components. Hence, we assessed the difference between octopus and background spectra using three criteria: (i) the difference between the accuracy of approximation of spectra from PCA, (ii) the difference between the low-dimensional spaces that spectra occupy, and (iii) the difference between the loci that octopus and background spectra occupy. It is important to note that PCs do not have specific meaning and do not correspond to particular aspects of colours. Chromatic aspects of colour depend on the shape of reflectance spectra rather than on their amplitude. To analyse the shape of reflectance spectra, we normalised them.

To analyse colours as they are seen through the eyes of potential predators, we use the receptor noise limited model (RNL) [34]. In aquatic habitats, colours change depending on depth and viewing condition due to the absorption and scattering of light in water [35,36]. Therefore, we model colours as they may be seen at the surface and at a depth of 10m when viewed from a close distance, as well as from further away to simulate the water veiling effect [36].

## 2. Materials and Methods

### 2.1. Experimental Setup and Data Collection

**Figure 1:**
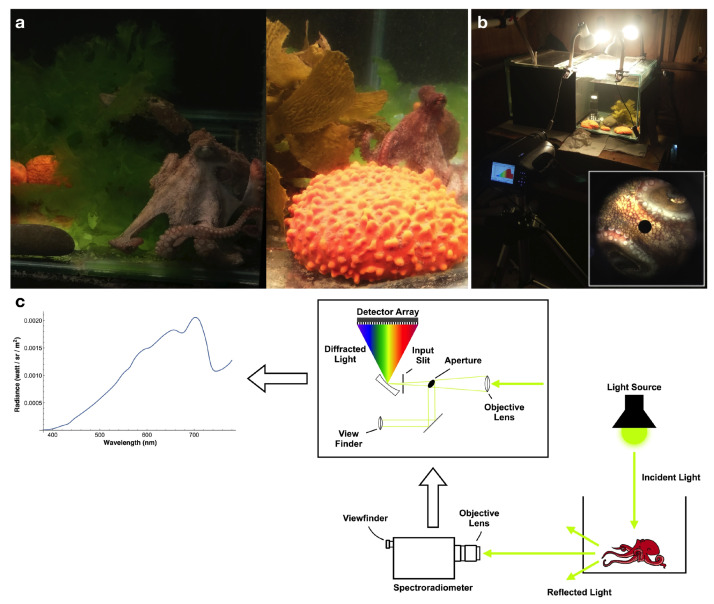
(**a**) Examples of octopus in tank with green algae, brown algae, and sponges. (**b**) Measurement setup—The spectroradiometer was placed on a tripod at 0.5 m from the tanks, and measurements were made through the glass. Inset shows an example of octopus skin as seen through the viewfinder of the spectroradiometer. The black circle at the centre indicates the area from which the measurement is taken. (**c**) Diagram detailing reflected light captured by the spectroradiometer and resulting radiance measurement. Incident light is reflected from an object and enters the spectroradiometer through the objective lens. The 1 aperture with a viewfinder allows measuring spectra of the selected area of the image of the object. The light is then diffracted and captured by a detector array, providing the output spectrum (inset is redrawn from PR-665 Spectrascan^®^ User Manual).

Six octopuses (*Octopus tetricus*) (160.45 ± 90.45 g) were captured in Hauraki Gulf, New Zealand, and placed in glass tanks of 90 cm × 45 cm × 40 cm (L × W × H) at the Leigh Marine Laboratory. Tanks were kept with constant seawater flow, filtered at 200 μm, from Goat Island Marine Reserve and provided with an additional aeration system. Octopuses were fed with live mussels every two days and held on a 12:12 h light:dark cycle. These tanks were divided into two compartments by use of a removable grey plastic sheet. One of them (the dwelling compartment) contained rocks, oyster shells, and a PVC pipe, all of which the octopuses could use for building dens. The other compartment (experimental side) contained colourful objects which are typically found in the octopus natural environment: green algae, brown algae, and orange sponges (see Figure 1a). Octopuses stayed in the dwelling compartment for approximately 1 week before we started to do spectral measurements. For spectral measurements of octopus skin, octopuses were moved to the side with the colourful objects, where they stayed for several hours. After the measurements, octopuses were returned to the dwelling compartment. Octopuses were released after experiments.

Spectra were measured from the octopuses and of the background objects (green algae, brown algae orange sponges) using a PR-655 SpectraScan^®^ spectroradiometer (Photo Research Inc., North Syracuse, NY, USA) (sampling range: 380–780 nm at 4 nm intervals). The spectroradiometer used is equipped with an objective lens and a viewfinder, allowing us to aim at our target and accurately define the measuring field within the field of view (see Figure 1b—inset and Figure S1). This enables us to make measurements without the need for a fibre probe to be in contact with the object, as the instrument can be used as a ’point-and-shoot’ camera, providing the advantage of being able to make measurements from a distance. For each radiance measurement, the spectroradiometer samples the light for a certain exposure time (based on light intensity), after which a dark current measurement is made for the same length of time, which is subtracted from the light measurement. Three consecutive measurements are done in this way, and the average of the spectra is provided by the instrument.

The spectroradiometer was placed at 0.5 m from the tanks and measurements were taken through the glass (Figure 1b). The acceptance angle of the spectroradiometer was 1. Since the distance to octopuses and background objects exceeded 0.5 m, the diameter of the circular area over which the spectral measurements were taken exceeded 0.9 cm, and, assuming that the refractive index of water is 1.35, for an object located in the middle of the tank the diameter of the area over which the spectral measurements were taken is 1.2 cm (see Figure 1c). The back wall and side walls of the tank were covered with black corrugated plastic. Measurement time could take between 1 and 18 s per point measurement, requiring the octopus to remain still. Any movement or change in body pattern resulted in aborting the current measurement. During measurements, the aeration system and all overhanging lamps were turned off, and tanks were illuminated from above with 4 incandescent lightbulbs (Philips Softone 100 W 240 V, Amsterdam, Netherlands). This arrangement of the illumination minimises the artefacts caused by reflections from the glass. Incandescent lightbulbs were chosen because they produce a smooth spectra curve, as opposed to fluorescent bulbs which have spikes at certain wavelengths and lack intensity at others. After measurements were taken, octopuses were moved back to the dwelling side of the tank.

Spectra of objects were divided by the spectrum of barium sulphate white standard placed inside the tank. This ratio is essentially a reflectance spectrum in the sense that the spectrum reflected from the object when it is illuminated by light of different spectral composition can be calculated as a product of this ratio and the illumination spectrum. We refer to this ratio as ’reflectance spectrum’. Note that this is not a true reflectance spectrum because the location of the standard is different from the location of objects. However, the tank environment had a relatively uniform illumination, and all measurements were made on surfaces which were nearly orthogonal relative to the angle of the spectrophotometer and which had no shadows cast over them. Therefore, the measured spectra reveal the properties of the object surfaces and not of illumination conditions. Another source of the error of measurements is a spectrally selective light absorption in water, which is prominent in the UV and far-red parts of the spectrum. To minimise the effect of light absorption we truncated the spectra at 700 nm. To evaluate the validity of this method, we recorded spectra of a colour standard (made from paint samples—Figure S2) covered by transparent plastic (laminated) together with the white standard located either at the same position as the colour standard or at different locations. The variation in the shape of spectra are small and attributed to addition of spectrally flat reflection due to specular component at the water-plastic interface (Figure S3). For more details, refer to Supplementary Materials—Colour Standard. Finally, the light is collected from a circular area of diameter 0.9–1.2 cm. Therefore, what was actually measured were the spectra averaged over this area. We took measurements from patches that appeared to be mostly uniform in their colouration, i.e., we did not measure mottled patterns of octopus skin.

To keep the white standard dry, a rubber o-ring was placed around it and was covered with transparent acrylic. Because of the light absorbance properties of the acrylic, corrections were made to the measured spectra. Due to low quantum flux below 400 nm the spectra were analysed in the range 400–700 nm (76 sampling points).

All animal handling and experiments were carried out under the approval of the University of Auckland Animal Ethics Committee (ref. 001761), and done in accordance with the Code of Ethics of the World Medical Association (Declaration of Helsinki).

### 2.2. Data Analysis

All data analysis was done in Wolfram Mathematica (version 11.3 for Mac OS X, Wolfram Research Inc., Champaign, IL, USA).

#### 2.2.1. Analysis of Spectra Similarity

To analyse the shape of the reflectance spectra, we normalised them by dividing each spectrum by the area under the spectral curve. Therefore, spectra were analysed twice: once for the non-normalised and once for the normalised spectra. A principal component analysis (PCA) was performed on the reflectance spectra of each group: octopus, green algae, brown algae, and sponges. For each group of spectra the covariance matrix was calculated as

(1) $$Cov\left(\lambda ,\mu \right)=\frac{\sum_{i=1}^{n}\left(\left(S_{i}\left(\lambda \right)-\bar{S(\lambda )}\right)\left(S_{i}\left(\mu \right)-\bar{S(\mu )}\right)\right)}{n}\;,$$

where *i* is the index corresponding to the measured spectrum, *n* is the total number of measured spectra for the given group, Cov is the covariance, λ and μ are wavelengths, $S_{i}\left(\lambda \right)$ is the *i*th spectrum, and $\bar{S(\lambda )}$ is the mean spectrum. Since there are 76 sampling points in the spectra measurements, the size of the covariance matrix is [76 × 76]. PCA was performed by finding eigenvectors (principal components) and eigenvalues through the ’Eigenvectors’ and ’Eigenvalues’ functions in Wolfram Mathematica. Spectra were reconstructed using the PCs as

(2) $$S_{i,m}^{a}\left(\lambda \right)=\bar{S(\lambda )}+\sum_{k=1}^{m}x_{i,k}E_{k}\left(\lambda \right)\;,$$

where *i* is the index corresponding to the measured spectrum, *k* is the index corresponding to the PC, *m* is the number of principal components used for the reconstruction, $S_{i,m}^{a}\left(\lambda \right)$ is the approximation of spectrum *i* using *m* principal components, $x_{i,k}$ is the coefficient (weight) for reconstruction of spectrum *i* using the *k*th PC, and $E_{k}\left(\lambda \right)$ is the *k*th eigenvector or PC. Note that eigenvectors are orthogonal and normalised to one. For each measured spectrum $S_{i}\left(\lambda \right)$, the coefficients for its reconstruction were calculated as:

(3) $$x_{i,k}=\int \;\left(S_{i}\left(\lambda \right)-\bar{S(\lambda )}\right)E_{k}\left(\lambda \right)\;d\lambda \;.$$

where integration is from 400 to 700 nm. This method of reconstruction of spectra provides the minimal root-mean-square error of reconstruction for a given group of spectra. The root-mean-square error (RMSE) of reconstruction of a spectrum $S_{i}\left(\lambda \right)$ using *m* principal components is given by:

(4) $$R_{i,m}=\sqrt{\frac{\int \;{\left(S_{i}\left(\lambda \right)-S_{i,m}^{a}\left(\lambda \right)\right)}^{2}\;d\lambda }{\Lambda }}\;,$$

where Λ denotes the range of integration.

The average RMSE was calculated as:

(5) $$\bar{R_{m}}=\sqrt{\frac{\sum_{i=1}^{n}{R_{i,m}}^{2}}{n}}\;.$$

A relative RMSE is defined as $\bar{r_{m}}=\sqrt{\frac{\bar{{R_{m}}^{2}}}{Y}}$, where *Y* is the total variance. When RMSE is calculated using the PCs from the same set of spectra it can expressed via eigenvalues. Let $y_{k}$ be the *k*th eigenvalue, then

(6) $$\bar{r_{m}}=\sqrt{\frac{\bar{{R_{m}}^{2}}}{Y}}=\sqrt{1-\frac{\sum_{k=1}^{m}y_{k}}{Y}}\;.$$

The number of PCs needed for reconstruction can be found from the dependence of the root mean square error of reconstruction $\bar{r_{m}}$ on the number of PCs.

#### 2.2.2. Comparison between Octopus and Background Spectra

(i) The difference between RMSEs calculated using the PCA from own and foreign groups of spectra is a measure of a difference between the groups of spectra because the average RMSE is minimal for the spectra, from which the principal components are derived. For each background group (green algae, brown algae, or sponge), reconstruction was performed using the mean octopus spectrum $S^{O}\left(\lambda \right)$ and the eigenvectors $E_{k}^{O}\left(\lambda \right)$ from octopus spectra, as well as the mean background spectrum $S^{B}\left(\lambda \right)$ and the eigenvectors $E_{k}^{B}\left(\lambda \right)$ from background spectra (Equations (2) and (3)) (index *O* corresponds to octopus and index *B* corresponds to background, i.e., green algae, brown algae or sponge). The average RMSEs were calculated using Equation (5) for both routines of reconstruction.

(ii) The difference between spectra was assessed as the difference between the subspaces that they occupy. The axes of the subspaces where the spectra lie are defined by the eigenvectors. The difference between eigenvectors can be assessed by examining their shape. However, because any linear combination of eigenvectors is also a vector belonging to this subspace the dissimilarity of eigenvectors does not imply that two subspaces are different. The subspaces are similar if any vector from one subspace can be closely approximates by linear combination of vectors from another subspace. To quantify the difference between the subspaces of octopus and background spectra, we calculated the maximum distance $d_{max}$ between a unity vector in one subspace and its projection onto the other subspace over all possible orientations. In the case of two dimensions, this distance is equal to the sine of the angle between the two planes. To calculate $d_{max}$ we use the following method (for a derivation see [37]—Appendix A). Consider a matrix W of cosines of angles between eigenvectors of the two spaces, which elements are calculated as

(7) $$w_{i,k}=\int \;E_{i}^{O}\left(\lambda \right)E_{k}^{B}\left(\lambda \right)\;d\lambda \;,$$

and a symmetric matrix $M={\mathrm{WW}}^{T}$ (where index ${}^{T}$ denotes transposed matrix). Let $L_{min}$ be the minimum eigenvalue of the matrix M, then the maximum distance is given by:

(8) $$d_{max}=\sqrt{1-L_{min}}\;.$$

Note that, because eigenvectors are orthogonal and normalised to one, if $E_{k}^{O}\left(\lambda \right)=E_{k}^{B}\left(\lambda \right)$ then W and M are unity matrixes, which implies that in the case of identical spaces $L_{min}=1$ and $d_{max}=0$.

(iii) To measure the difference between octopus and background spectra, we consider spectra as points in the *m*-dimensional space, where the coefficients $x_{i,k}$ (Equation (3)) are plotted along the coordinate axes. The Fisher discriminant analysis finds a direction in a multi-dimensional space along which the two groups of data are best separated and finds the distance along this line as a ratio of the difference of the means to the standard deviations of the spread. Let

(9a) $${x_{k}}^{O}=\frac{1}{n}\sum_{i=1}^{n}{x_{i,k}}^{O}\;,$$

(9b) $${x_{k}}^{B}=\frac{1}{n}\sum_{i=1}^{n}{x_{i,k}}^{B}$$

be the mean of loci of octopus and backgrounds spectra, respectively, and

(10a) $${K_{k,j}}^{O}=\frac{1}{\left(n-1\right)}\sum_{i=1}^{n}\left({x_{i,k}}^{O}-\bar{{x_{k}}^{O}}\right)\left({x_{i,j}}^{O}-\bar{{x_{j}}^{O}}\right)\;,$$

(10b) $${K_{k,j}}^{B}=\frac{1}{\left(n-1\right)}\sum_{i=1}^{n}\left({x_{i,k}}^{B}-\bar{{x_{k}}^{B}}\right)\left({x_{i,j}}^{B}-\bar{{x_{j}}^{B}}\right)$$

be the elements covariance matrices of octopus and background spectra, respectively. Let $\bar{X^{O}}$ and $\bar{X^{B}}$ be vectors with elements given by Equation (9a) and (9b), respectively, and $K^{O}$ and $K^{B}$ be covariance matrices with elements given by Equation (10a) and (10b), respectively. Note that when the PCA is performed on the set of spectra that are plotted as points in the *m*-dimensional space, the mean $\bar{X}=0$ and covariance K is a diagonal matrix. The Fisher discriminant distance is given by

(11) $$F\left(O,B\right)=\sqrt{{\left(\bar{X^{O}}-\bar{X^{B}}\right)}^{T}{\left(K^{O}+K^{B}\right)}^{-1}\left(\bar{X^{O}}-\bar{X^{B}}\right)}\;,$$

where index ${}^{-1}$ denotes inverse and ${}^{T}$ denotes transpose.

#### 2.2.3. Modelling of Colours

**Figure 2:**
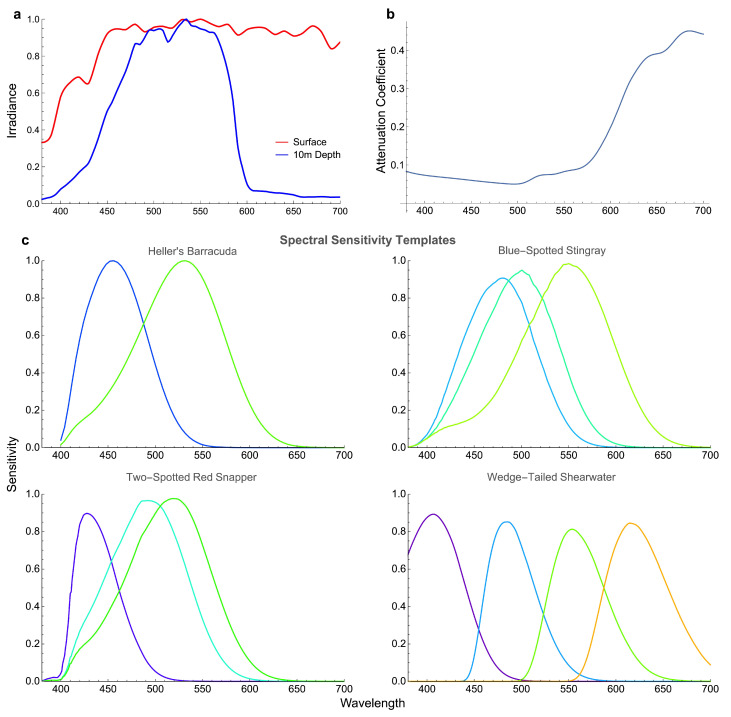
(**a**) Illumination profiles used for colour modelling at surface (red) and 10m depth (blue) [46], both divided by their respective maximum (normalised to 1). (**b**) Attenuation coefficient from absorption and scatter of water used for colour modelling at 10m depth [46]. (**c**) Sensitivity templates for four potential octopus predators: Heller’s barracuda, blue-spotted stingray, two-spotted red snapper, and wedge-tailed shearwater. The colour of each curve corresponds to the wavelength of maximum absorbance for the visual pigment (see Table 1). Note that the sensitivity curves for some cones do not reach 1, as they are first normalised and then transformed using the ocular media transmission spectrum.

The spectral sensitivities were modelled using A1 templates of visual pigment absorption [38], equations of bird oil droplet transmission [39], and available data on ocular media transmittance. The parameters used for modelling are given in Table 1 and spectral sensitivities are shown in Figure 2. The receptor quantum catches were calculated for different depths and distances to the objects as described in [36]. Let $R_{i}\left(\lambda \right)$ be the spectral sensitivity of a receptor of a spectral type, then quantum catch $Q_{i}$ of a receptor is given by

(12) $$Q_{i}=\int \;G_{s}\left(\lambda ,z\right)R_{i}\left(\lambda \right)\;d\lambda \;,$$

where integration is done in the range 400–700 nm and $G_{s}\left(\lambda ,z\right)$ is the light spectrum entering the eye, which depends on the distance *z* from the viewer to the object. The integration range excludes the UV part of the spectrum due to the limitation of the method. This may lead to an error in the calculation of quantum catch for the wedge-tailed shearwater, which has a violet-sensitive photoreceptor whose sensitivity extends to the UV part of the spectrum (Figure 2c). However, because water absorbs UV light strongly (Figure 2), the truncation of the spectrum at 400 nm is unlikely to have a significant effect on the calculated quantum catch. To estimate the inaccuracy due to truncation of the spectrum, we calculated the quantum catch of the wedge-tailed shearwater violet-sensitive cone using both the spectral range that includes UV (300–700 nm) and the range that excludes it (400–700 nm). We used 146 spectra of bird-dispersed fruits which have been previously used to assess differences in colour perception between UV- and violet-sensitive birds [40]. The quantum catches were calculated both for surface illumination and spectrum measured at 10m depth [41]. For the violet-sensitive receptor of the wedge-tailed shearwater, the error in the quantum catch calculations with and without UV is only 0.04 of the total quantum catch at 10m depth, and 0.17 at the surface.

Because light is absorbed and scattered in water, the light spectrum depends on the distance from the object, which leads to the water veiling effect [42]. The equation used for modelling the veiling effect is identical to that used in earlier publications [36,41]. The spectrum of light entering the eye can be expressed as a weighted sum of a spectrum corresponding to the zero distance and the spectrum corresponding to the distance of infinity as (an excellent derivation of this equation is given by Johnsen [42]):

(13) $$G_{s}\left(\lambda ,z\right)=S\left(\lambda \right)I\left(\lambda \right)\exp\left[-a\left(\lambda \right)z\right]+I^{0}\left(\lambda \right)\left(1-\exp\left[-a\left(\lambda \right)z\right]\right)\;,$$

where I(λ) is the illumination spectrum, S(λ) is the reflectance spectrum of an object, $I^{0}\left(\lambda \right)$ is the light spectrum for an object located at infinity or absent, and a(λ) is the beam attenuation coefficient, which depends on absorption and scattering of light in water [36,41] (Figure 2). We performed calculations for the case of illumination by horizontal light and viewing objects in the horizontal direction [41]. In this case, the spectrum of illumination is identical to the light spectrum in the absence of the object, i.e., $I\left(\lambda \right)=I^{0}\left(\lambda \right)$. The illumination spectrum I(λ) and the beam attenuation coefficient a(λ) are from Marshall and Vorobyev [41]. The illumination spectra were measured at several depths with a horizontally oriented spectroradiometer, while the attenuations coefficient was obtained from measurements made at 10m depth. It is important to note that Marshall and Vorobyev [41] report the illumination spectra and the attenuation coefficient obtained in clear water on coral reef. Therefore, the illumination spectra and attenuations can differ substantially in conditions of less clear water. We also report results for objects located at the surface. In this case, we assume that objects are illuminated by the standard D65 daylight [43] and ignore the veiling effect.

To model colour discrimination, we use a logarithmic version of the RNL model and a chromaticity diagram based on this model [44]. Photoreceptor signals were calculated as:

(14) $$f_{i}=\ln\left(Q_{i}\right)\;,$$

where i=L,M,S,VS, corresponding to ’long’, ’medium’, ’short’, and ’very short’ wavelength sensitive receptors, respectively. The chromaticity coordinates for dichromatic, trichromatic and tetrachromatic visual systems were calculated using equations from Kelber et al. [44] (see Equation (S1a–c)).

In all cases it is assumed that the Weber fraction for the long wavelength cone is 0.05, and for the other cones they are obtained from cone counts [34]. The Weber fractions used were as follows: barracuda [0.1, 0.05] (S, L), stingray [0.1, 0.07, 0.05] (S, M, L), snapper [0.1, 0.07, 0.05] (S, M, L), and bird [0.1, 0.07, 0.07, 0.05] (VS, S, M, L). The Euclidian distance in this space describes the discriminability of colours. If the distance between colours in chromatic space is smaller than a certain threshold, then the colours cannot be discriminated and there is a colour-match for this particular visual system. Two methods for quantifying the difference between octopus and background colours were used: (i) the Fisher discriminant distance as defined by Equations (9)–(11), and (ii) the index of indiscrimination, which indicates the percentage of colours from one set that are indiscriminable from any colour from the second set for a particular visual system given certain illumination conditions [45]. For octopus and background spectra the locations in the RNL chromatic diagrams were evaluated, and the colour distance between octopus and background loci were calculated as:

(15) $$\Delta S^{i,k}=\sqrt{\sum_{j}^{m}\left(X_{j}^{Oi}-X_{j}^{Bk}\right)}\;,$$

where $X_{j}^{Oi}$ and $X_{j}^{Bk}$ are the *j*th coordinate of the *i*th octopus colour and *k*th background colour, respectively. If the distance $\Delta S^{i,k}$ is smaller than a given threshold, then the octopus colour can be assumed to match the background colour. For each octopus colour, the distances to all background colours were calculated, and if this distance for at least one colour was smaller than the threshold, then this colour was assumed to colour-match the background, and the index of indiscrimination was evaluated as the proportion of octopus colours that match background colours.

## 3. Results

Octopus responses varied when transferred to the experimental side of the tank: although sometimes octopuses adopted colours that were similar to those of backgrounds, this was not always the case—i.e., octopus colours were cryptic at times. Usually octopuses displayed uniform colouration. Occasionally, they adopted mottled patterns. We did not measure spectra of octopus when they adopted mottled patterns

### 3.1. Reflectance Spectra

**Figure 3:**
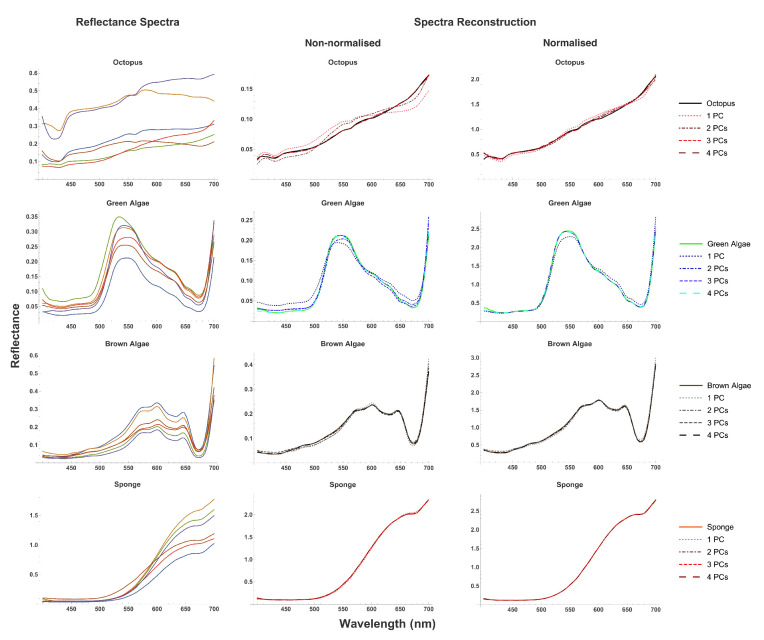
Examples of reflectance spectra (**left**) of octopus, green algae, brown algae, and sponge, and their reconstruction from PCA using 1–4 principal components (solid line is original spectra, dotted/dashed lines are reconstructions) for both non-normalised (**centre**) and normalised (**right**) spectra.

A total of 192 octopus spectra, 107 green algae spectra, 107 brown algae spectra, and 107 red-orange sponge spectra were obtained. The reflectance curves for green and brown algae have peaks at approximately 530 nm and 600 nm, respectively, (see Figure 3). Sponge spectra have a significant increase in reflectance from around 550 nm to 650 nm. Furthermore, above 600 nm the sponge reflectance intensity becomes higher than that of the white standard. Most octopus spectra had peaks towards the long-wavelength end of the visual spectrum. However, 13 octopus spectra had peaks between 516 nm and 580 nm. As these peaks are much shallower than those in the spectral curves of algae, all octopus spectra can be distinguished from algae spectra by their shape. The overall range of reflectance of octopus is broader than that of algae and sponge (see Figure 3).

In order to reduce the dimensionality of spectra, PCA was carried out with non-normalized and normalised spectra. We reconstructed spectra using a PCA (Figure 3) and calculated the root mean square error of reconstruction as a function of the number of PCs (Figure S4), while many spectra can be reasonably approximated using three PCs, improvement in accuracy of reconstruction of both normalised and original spectra becomes considerably smaller after the fourth principal component (see Figure S4-left), indicating that four PCs are the minimum required for accurate representation of spectra.

**Figure 4:**
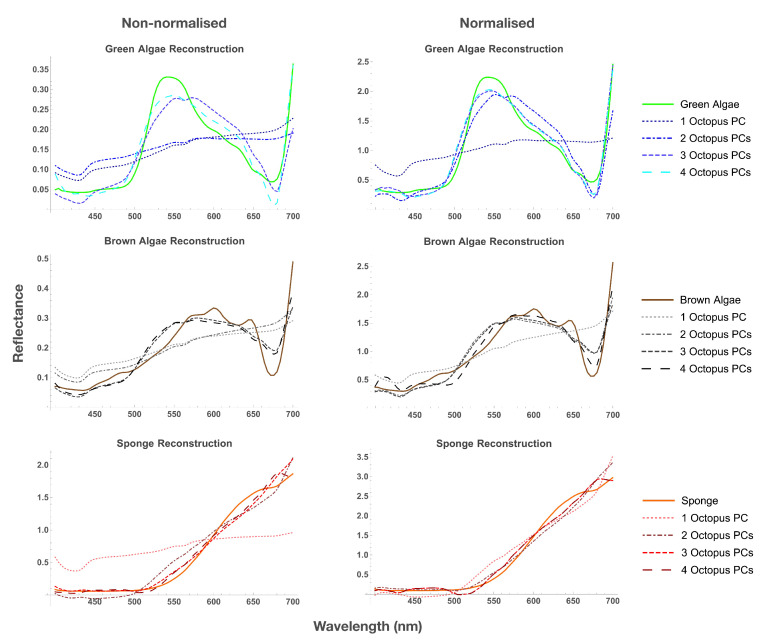
Examples of reconstruction of spectra of green algae, brown algae, and sponge, using 1–4 PCs of octopus (solid line is original spectra, dotted/dashed line is reconstruction) for non-normalised (**left**) and normalised (**right**) spectra. When four PCs were used, reconstruction of normalised spectra was more accurate than the reconstruction of non-normalised spectra.

To find out whether the spectra of backgrounds can be represented using the principal components of octopus (see Figure 4), we estimated the accuracy of reconstruction of background spectra using octopus PCs (Figure S5). The error of reconstruction of background spectra with octopus PCs was significantly greater than with own PCs indicating that PCs of octopus and background spectra differ substantially.

To quantify the difference between subspaces occupied by octopus and background spectra, we estimated the maximal distance between unity vectors belonging to these spaces ($d_{max}$, see Equations (7) and (8)) for one-, two-, and three- dimensional subspaces [37]. In the case of identical subspaces (i.e., any spectrum can be represented as a linear combination of spectra from the other subspace), this distance is equal to 0; in the case of completely different subspaces (i.e., the subspaces are orthogonal to each other), this distance is equal to 1. The distances obtained were substantial (see Table 2): in the case of the three dimensional subspaces, the distances for non-normalised spectra were equal to 0.66, 0.74, and 0.7 for green algae, brown algae, and sponge, respectively, while the normalised spectra showed differences of 0.8, 0.88, and 0.59, respectively. These values indicate that octopus and background reflectance spectra belong to subspaces that are nearly orthogonal to each other and, hence, the spectra are significantly different.

**Figure 5:**
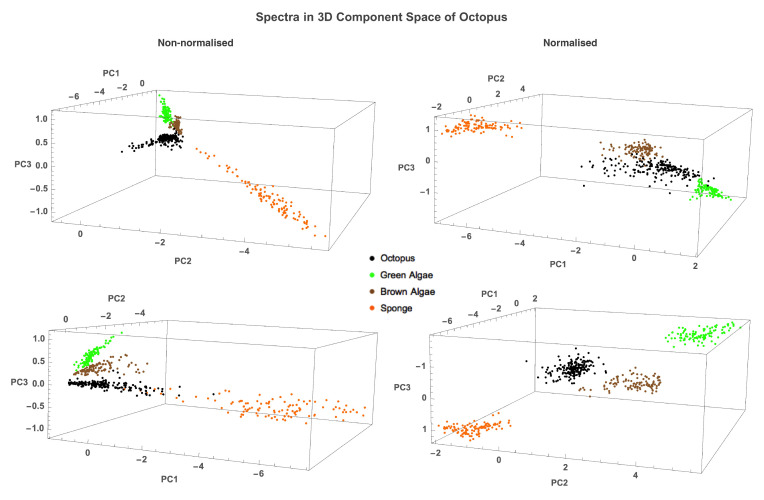
Two different viewing angles of loci of octopus (black), green algae (green), brown algae (brown), and sponge (orange) spectra in the three-dimensional principal component space of octopus for non-normalised (**left**) and normalised (**right**) data. Octopus spectra occupy loci that are significantly different from that of backgrounds. Normalisation increased the distance between spectra groups, as evidenced by the size of the PC axes.

The PCA allows us to represent spectra as points in an *m*-dimensional space, where *m* is the number of principal components, facilitating comparison between the spectra. Background spectra were placed in the 3D principal component space of octopus to compare the spread of values of the two groups between different principal components [37]. The amount of overlap between the values of two datasets suggests a degree of similarity between the spectra of said groups. It is clear that, for both non-normalised and normalised data, octopus, green algae, brown algae, and sponge spectra mostly occupy very distinct loci, suggesting that each group is significantly different (Figure 5).

To quantify the difference between octopus and background spectra, we calculated the Fisher discriminant distance between their loci (Equation (11)) A unity distance in this representation corresponds to one standard deviation. The difference between non-normalised octopus and background spectra using 3 PCs was 4.14, 3.11, and 4.21 for green algae, brown algae, and sponge, respectively. Overall, normalisation increased distance between subspaces: 6.76, 4.08, and 7.38 for green algae, brown algae, and sponge, respectively, (see Table 3). It is therefore likely that any proximity between octopus and background points seen in the non-normalised octopus PC space is due to amplitude rather than shape of spectra.

### 3.2. Octopus in the Eyes of Different Animals

**Figure 6:**
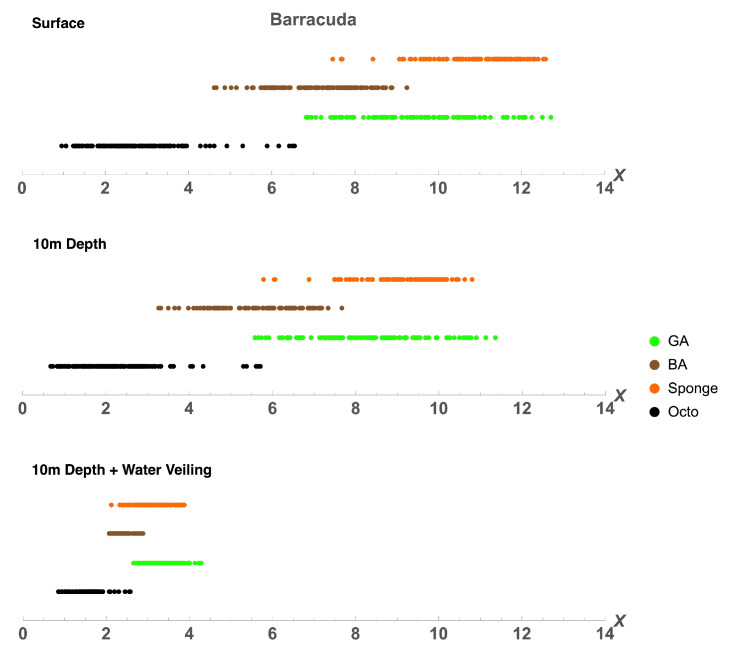
Chromaticity diagrams of barracuda showing chromatic coordinates of octopus (black), green algae (green), brown algae (brown), and sponge (orange) colours at surface, 10m depth, and 10m depth plus water veiling. Depth and water veiling allow for a small overlap between octopus colours and those of green algae and sponge. The distance between clusters is reduced with increased depth and viewing distance (water veiling).

**Figure 7:**
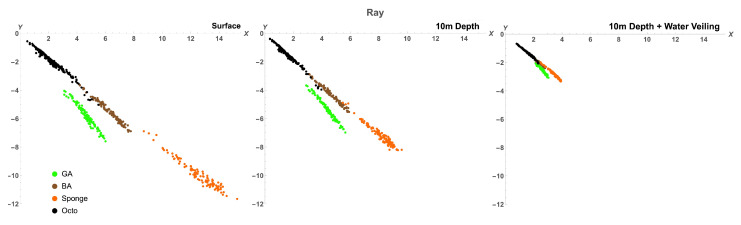
Chromaticity diagrams of the blue-spotted stingray showing chromatic coordinates of octopus (black), green algae (green), brown algae (brown), and sponge (orange) colours at surface, 10m depth, and 10m depth plus water veiling. A slight overlap between octopus and brown algae colours can be seen in all three viewing conditions, but no overlap can be seen with either green algae or sponge colours without the effect of water veiling. Water veiling allows for a small overlap between octopus and green algae colours. The distance between clusters is reduced with increased depth and viewing distance (water veiling).

**Figure 8:**
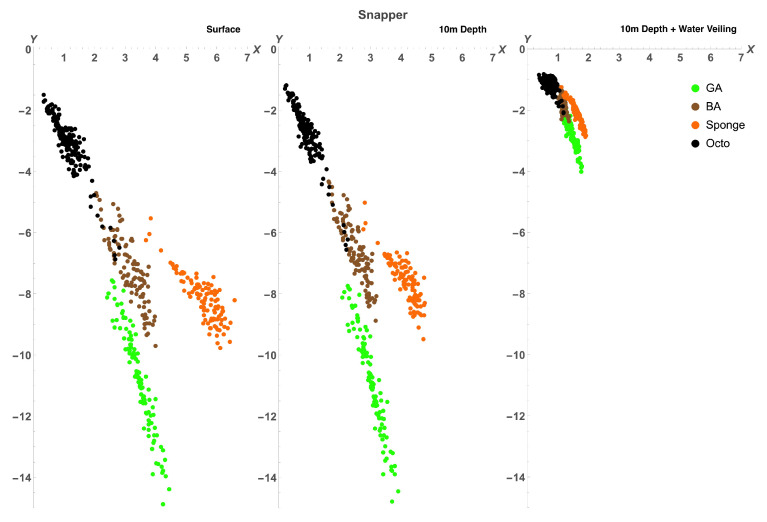
Chromaticity diagrams of the two-spotted red snapper showing chromatic coordinates of octopus (black), green algae (green), brown algae (brown), and sponge (orange) colours at surface, 10m depth, and 10m depth plus water veiling. Overlap between octopus and brown algae colours can be seen in all three viewing conditions, but no overlap can be seen with either green algae or sponge colours without the effect of water veiling. Water veiling allows for a small overlap between octopus and green algae colours. The distance between clusters is reduced with increased depth and viewing distance (water veiling).

**Figure 9:**
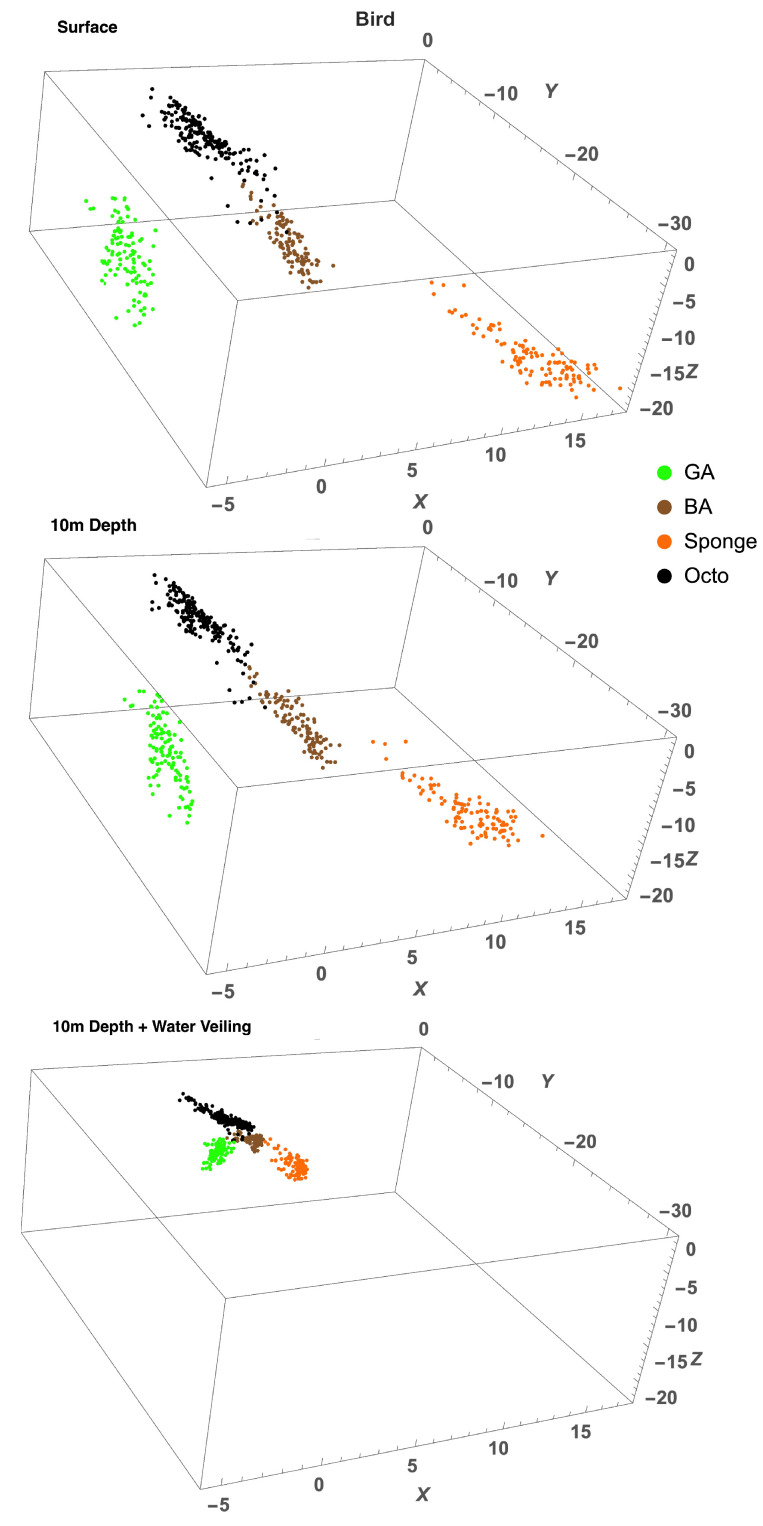
Chromaticity diagrams of the wedge-tailed shearwater showing chromatic coordinates of octopus (black), green algae (green), brown algae (brown), and sponge (orange) colours at surface, 10m depth, and 10m depth plus water veiling. There is no overlap between octopus and green algae or sponge colours at any viewing condition. A slight overlap between octopus and brown algae can be seen when water veiling is added. The distance between clusters is reduced with increased viewing distance (water veiling).

To find out if the range of variability of octopus spectra allows it to be cryptic in the eyes of potential predators, we plotted the colours of octopus, green algae, brown algae, and sponge in the chromaticity diagrams (Equation (14)) of four different species: a dichromatic fish (barracuda, *Sphyraena helleri*), two trichromatic fishes (blue-spotted stingray, *Neotrygon kuhlii*, and two-spotted red snapper, *Lutjanus bohar*), and a tetrachromatic bird (wedge-tailed shearwater, *Puffinus pacificus*). The diagram is based on a receptor noise model of colour discrimination [34,36]. Note that this method ignores the lightness direction in the colour space and, hence, reduces the dimensionality of colour representation. Therefore, colours for tetrachromatic, trichromatic, and dichromatic visual systems are plotted in three-, two- and one- dimensional chromatic diagrams, respectively. Colours were modelled (Equations (12) and (13)) as seen at the surface (D65 illumination) and at a depth of 10m from close distance and from a distance of 5 m to simulate the veiling effect caused by light absorption and scatter in water Figure 6, Figure 7, Figure 8 and Figure 9.

For all visual systems and viewing conditions, the cluster of green algae colours does not overlap with the cluster of octopus colours, except for the dichromatic fish, for which there is a very slight overlap at 10m depth (Figure 6). For the dichromatic fish, the cluster of brown algae colours strongly overlap with octopus colours at all viewing conditions; for the two trichromatic fishes, the cluster of brown algae colours overlaps slightly with octopus colours, increasing with the effect of water veiling (Figure 7 and Figure 8); and, for the tetrachromatic bird, brown algae colours do not overlap at any viewing condition (Figure 9). For all visual systems and viewing conditions, the cluster of sponge colours does not overlap with the cluster of octopus colours, except for the dichromatic fish, for which there is a small overlap when considering the effect of water veiling (Figure 6). To quantify the separations between colour loci we calculated the Fisher discriminant distance (Equation (11)) (measured in standard deviations). This measure considers the spread of colours but does not take into account discriminability of colours by visual systems (Table 4). The Fisher separation is substantial for all visual systems, with minimal separation for brown algae (∼3σ) across all visual systems, followed by green algae for dichromatic and trichromatic fish (∼4.5σ). The separation is maximum for the bird visual system (∼7σ for green algae and sponge). Note that the separations do not strongly depend on viewing conditions because the changes in illumination and veiling have similar effect on the difference between means of clusters of colours and on the standard deviation

To estimate the ability of octopus to match colours of backgrounds, we used a receptor noise limited model of colour discrimination [34,36] and calculated the percentage of octopus colours that cannot be discriminated from colours of algae and sponge (see Table 5). For each octopus colour we calculated the distance to background colours, and if the distance was less than a given threshold for at least one background colour, then the octopus colour was assumed to be indiscernible from a given type of background. The method is identical to that used by Osorio and Vorobyev [45] to calculate the percentage of fruit colours indiscernible from foliage colours for a primate visual system [45]. It is important to note that for visual systems used in this study, the value of the threshold is not known. Moreover, threshold values may vary depending on viewing conditions, attention, and motivation [36,52]. Therefore, we performed calculations for the three values of thresholds corresponding to distances in the chromatic diagram of 0.5, 1, and 1.5.

Our calculations suggest that octopus can match brown algae for the visual systems of dichromatic and trichromatic fish, as well as for a tetrachromatic bird. Only for a dichromatic fish, barracuda, all three types of background can be matched for all viewing conditions (with the exception of 0.5 threshold at the surface). For the visual system of stingray octopus can better match green algae than sponge, but for the visual system of snapper it matches sponge to a higher degree than green algae. Very few sponge and green algae colours can be matched for the bird visual system, and for sponge only with the help of water veiling. The percentage of octopus colours that are indiscernible from backgrounds increases with the increase of thresholds. Depth has little effect on the colour-match; only in the case of brown algae viewed by barracuda an obvious increase in the colour-match with the change of depth from surface to 10m is observed. The effect of viewing distance is consistent for all visual systems and background objects - the percentage of indiscernible colours is maximal when octopus and backgrounds are viewed from a distance, because water veiling decreases saturation of colours and therefore decreases the distance between them in the chromatic diagram.

To avoid being eaten, octopuses needs to be cryptic in the eyes of several predators. To quantify the potential of octopus to be simultaneously cryptic for different predators, we counted the number of octopus colours that are indiscernible from backgrounds for pairs of visual systems and calculated a ratio of such colours to colours indiscernible for one visual system. If this ratio is equal to one, then all colours that are cryptic for one visual system are also cryptic for another visual system. Table 6 presents the overlap ratio calculated at surface, 10m depth, and 10m depth plus water veiling using threshold values of 0.5, 1, and 1.5. It can be appreciated that overlap is not strongly affected by depth.

In the case of green algae, practically all colours that are cryptic for snapper are also cryptic for stingray and for barracuda. Likewise, almost all colours which are cryptic for barracuda at depth are also cryptic for stingray (80–100%), but are mostly only cryptic to snapper with the effect of water veiling (95–100%, as opposed to 0–29% without water veiling). However, colour-matching for stingray does not imply colour-matching for other fish in most cases. As previously mentioned, green algae colours cannot be matched for the tetrachromatic bird except when water veiling is taken into account. Then, all colours that are cryptic to the bird are cryptic to all fish.

In the case of brown algae, all colours that are indiscriminable to the visual system of the shearwater are also indiscriminable to the ray, snapper, and barracuda. On the other hand, most octopus colours that match brown algae colours for the dichromatic visual system of barracuda can be discerned by a tetrachromatic bird, and many can be discerned by trichromatic fish (61–72% at 10m depth), although water veiling helps the octopus in becoming considerably more cryptic in their eyes. Most colours which match brown algae for stingray are also matched for the other two fish (76–100%, depending on viewing conditions and threshold value), and many of the octopus colours which are indiscernible from brown algae for snapper are also indiscernible for stingray and barracuda. However, most of these octopus colours will be distinguished by the shearwater.

In the case of sponges, colours that are indiscriminable for stingray are only indiscriminable for barracuda and snapper with the effect of water veiling. Most octopus colours that match sponge colours for the dichromatic visual system of barracuda are also matched for snapper, but none of them are matched for stingray when no water veiling is considered. Many colours which are cryptic for snapper are also cryptic for barracuda, but almost all of them are conspicuous for stingray. As previously mentioned, sponge colours can only be matched for the tetrachromatic bird with help from the effect of water veiling and assuming a threshold value of 1.5. In this case, all colours indiscriminable for the shearwater are also indiscriminable for all three fish species.

## 4. Discussion

Our results show that octopus spectra, regardless of whether they are normalised or not, are significantly different from those of the tested background objects: green algae, brown algae, and sponge. We demonstrated that (i) background object spectra cannot be accurately reconstructed from the first 4 principal components of octopus spectra (Figure 4), (ii) the subspaces occupied by octopus and background object spectra differ substantially (Table 2), and (iii) octopus and background object spectra occupy distinct loci in the octopus principal component space (Figure 4 and Figure 5). The difference between spectra does not necessarily mean that the colours corresponding to these spectra are different (light stimuli having different spectral composition but having identical colours are called metameric stimuli, [43]). However, stimuli that are metameric for one visual system may differ in their colour for another visual system. Therefore, while octopus can be cryptic for one visual system, other visual systems can break the camouflage of octopus. It is important to note that the range of variability of the overall reflectance of octopus spectra significantly exceeds the range of variability of each of background spectra (Figure 3) Therefore, octopus can be cryptic for monochromats, such as other cephalopods [10,11] or marine mammals [53,54,55].

The clusters of octopus colours, as they are seen through the eyes of potential predators, are also clearly different from the clusters of background colours (Figure 6, Figure 7, Figure 8 and Figure 9, Table 4). However, some octopus colours may be similar to those of brown and green algae and orange sponges in the eyes of dichromatic and trichromatic fish, and to brown algae in the eyes of a tetrachromatic bird. Hence, the range of octopus colours may allow it to be cryptic in the eyes of potential predators.

It is important to note that the range of variability of octopus colours can be greater than we observed. As there were no predators in our tanks, it is possible that the motivation to change colour to become cryptic was reduced, while relatively newly-captured octopuses were more likely to change colour in an attempt to blend in with their new surroundings (as were smaller octopuses compared to larger ones), we observed that, with time, octopuses became more active and spent more time moving around the tank, indicating that they were habituated to their environment and were aware of the lack of dangers in the tank. Therefore, it cannot be ruled out that the measured octopus spectra differed strongly from green/brown algae and sponge spectra due to an absence in motivation rather than the lack of capability. While behaviour studies in captivity provide many advantages (e.g., being able to control variables such as the lighting conditions and the nature (and colour) of the objects in the octopus’s immediate environment, as well as facilitating data collecting with the spectrophotometer), there are also shortcomings that have to be considered. Because spectra were measured from octopuses in a non-natural environment, there are inherent limitations to this study—mainly, that behaviour of animals in captivity differs from that of animals in their natural habitat, which will be reflected in the results obtained. Taking measurements in their natural environment or increasing the motivation of octopuses to camouflage in the tank might allow us in the future to measure octopus spectra that are more similar to those of the objects around it.

Furthermore, the actual colours of octopus can be more saturated than those we have reported due to inherent limitations in the method of recording spectra. There is no single reflectance spectrum for an object: a single spot on a surface will have numerous reflectance spectra which depend on both the angle of incident light and the angle of observation [35]. We measured spectra over a patch with diameter of 0.9–1.2 cm (see Section 2). Sometimes, the octopus colouration did not appear uniform within the patch. Since we measured the spectra averaged over the patch, the recorded spectra correspond to less saturated colours than those that octopus can adopt [12,13]. However, this limitation of the method is unlikely to significantly impair our estimate of the ability of colour camouflage of octopus because when a predator observes an octopus from a reasonable distance, the predator eyes would also average colours over patches of octopus skin. It is also important to note that calculations of colours for a tetrachromatic bird which has a violet-sensitive cone with sensitivity extending to the UV part of the spectrum are not fully accurate due to limitations in the methods—we were not able to measure in the UV part of the spectrum. However, the error arising from this is only 0.04 for calculations at 10m depth. This error is marginal compared to the 0.1 Weber fraction of the violet-sensitive cone. One needs to be more careful with the results reported for the surface, where the error is 0.17, which exceeds the 0.1 Weber fraction.

Many other examples of camouflage observed in nature do not rely on spectra matching (e.g., [56,57,58,59,60]); instead, the colour match is achieved for a particular visual system. It may therefore not be surprising that octopuses do not match the reflectance spectra of surrounding objects. An example of an animal that matches the background spectrum is the diamond weevil, which matches the reflectance spectra of green leaves [61], allowing for effective camouflage for any visual system. However, the diamond weevil only matches the reflectance spectrum of green backgrounds, which can limit its range and behaviour to that specific background. On the other hand, an octopus needs to match many backgrounds and it would be unrealistic to expect that it can match all of them for all visual systems.

Analysis of our chromaticity diagrams suggests that octopuses can match the colours of all objects tested for dichromatic visual systems (albeit with considerable differences in the degree of resemblance), approximate algae colours for trichromatic visual systems, and resemble brown algae colours for a tetrachromatic visual system. Water veiling evidently increases colour-matching by reducing contrast and altering the appearance of colour [36]. It is important to note that colours were modelled as seen from ideal conditions (surface at close distance), at 10m depth, and with 5 m of water veiling effect, but potential predators could be much further away, while octopuses are mostly benthic organisms and seldom swim at the surface, they can sometimes be found close to the surface in tide pools. In these cases, however, there are no predatory fish around, and only the calculations for the shearwater are ecologically relevant. Therefore, it is important to consider that calculations for the surface should be used mainly for comparison with the other viewing conditions rather than for understanding octopus camouflage itself. On the other hand, the condition of viewing from close distance at depth is relevant, as instances have been recorded in which predatory fish failed to detect cryptic octopuses while swimming past them repeatedly at very close range (up to 15 cm), illustrating the effectiveness of octopus camouflage [62]. With increasing viewing distance the water veiling effect will be much stronger, making the octopus colours even more difficult to discriminate from the background ones. Furthermore, colours were modelled in clear water. With added turbidity, the attenuation coefficient would be greater, and colour distance would decrease. It is important to emphasise that we are only analysing octopus camouflage in terms of background colour-matching. Changes in skin texture and body position/orientation also have a strong effect on camouflage effectiveness and will decrease the probability of discrimination even further. Potential predators may use other sensory cues and do not rely exclusively on vision for hunting, but this is outside the scope of this study.

It is clear that chromatic similarity between octopus and brown algae exists across all visual systems modelled—after all, the chromatophores in the octopus skin range from yellow-orange through to red, brown, and black [15,63]. This may be a reason why octopuses prefer to imitate specific or key features of select objects in their surroundings [18]. It is possible that octopuses choose objects which they can resemble closely enough to fool most predators. Indeed, it has been pointed out that for colour camouflage to be effective in a complex environment, individuals would need to choose backgrounds which are similar to their appearance [64], a behaviour which has been observed in some species (e.g., [65]). While octopuses can manipulate their appearance and therefore are not as restricted by their surroundings for camouflage compared to other taxa, their abilities are not without limit, and it is entirely possible that some objects are generally easier to match than others.

Animals with different visual systems ranging from monochromatic through to tetrachromatic predate on octopus. Therefore, it may be simple to hide from some, but might prove more difficult to fool others. It has been observed that animals can change their body pattern or colour in response to particular predator types (e.g., [2,66,67,68,69,70]). At least some octopus species apparently choose particular body patterns of varying levels of crypsis or mimicry depending on their judgement of perceived threats [2,68]. Cuttlefish show specific displays (body patterns varying in level of conspicuousness) and anti-predator behaviours when in the presence of some predators, but not others, and vice versa [66,67]. Chameleons, under identical viewing conditions, have been observed to show body patterns varying in both chromatic and achromatic elements depending on whether snakes or birds are present, and yet appear fairly well camouflaged to both visual systems. This may in part be determined by the viewing angle of predators: snakes see chameleons from below (against a high illumination background), whereas birds see them from above (against a low illumination background) [69,70]. Our results show that while some body colours allow the octopus to camouflage against multiple predators, others are predator-specific, and only a select few will successfully fool all predators modelled. This varies considerably depending on the colours of the background objects. We did observe that the odds of an octopus fooling a particular predator by using a particular colour are higher if that same colour fools a predator with more visual pigments. This gives the octopus the advantage of being able to choose a particular body pattern and increase the odds of being effectively camouflaged for many different predator types, at least against certain backgrounds. Furthermore, viewing distance can play an important role in some cases by considerably increasing the probability of a particular octopus colour being cryptic for more than one visual system simultaneously. However, the overlap is far from absolute, and thus the octopus would need to switch between different colours to hide from particular predators. Octopus camouflage is certainly not universal but is without a doubt adaptable.

How can a colour-blind animal such as the octopus achieve colour-matching? One possibility is that in its environment colour is directly related to the nature or texture of the object and, rather than colour-matching, octopuses attempt to match the object itself. Octopuses would need to have an innate knowledge that grass is green, sand is yellow, etc., and then adjust the brightness accordingly. This would explain why in experiments where cuttlefish were placed on blue or red sand [14] or gravel [71], they responded to changes in brightness but were unable to match the colour, as these are substrates which they would never find in their natural environment. Another possibility is that colour is directly related to brightness, and the colour-blind octopuses only need to respond to the brightness cues. Once again, this would have to be limited to the natural colours existing in the octopus’s environment. A similar behaviour has been observed in tree frogs which, even though they possess colour vision [72,73], change between green and brown body colouration based on background brightness and not hue [65,74].

Visual opsins have been found in the skin of different cephalopods, and thus it has been suggested that they might have dermal light-sensing capabilities [75,76]. Whereas this mechanism for extraocular light sensing could help cephalopods regulate body colours and patterns, so far only one opsin type has been found. It has been shown that the spectral sensitivity of the opsin in their skin is essentially identical to the spectral sensitivity of the visual pigment in their eyes [76,77], meaning that light sensing through their skin would also be monochromatic. Colour discrimination would therefore be unlikely, although it could aid in brightness matching. Nevertheless, the presence of other opsins with different spectral sensitivities cannot be ruled out. Furthermore, because skin opsins are associated with chromatophores [75] and possibly iridophores, it is possible that these organs could act as “spectral tuning filters”, allowing the dermal opsins to receive wavelength information [76]. A more recent hypothesis [78] suggests that octopuses could obtain colour information from their environment thanks to a strong chromatic aberration (a phenomenon which occurs because the refractive index of a lens varies with wavelength [43,79]). The horizontal, off-axis pupil of octopuses actually enhances said chromatic aberration [78,80] and, because there is a strong relationship between spectrum, distance, and focus, it is possible that this amplified blur provides spectral information through the focus range of a scene: different colours will be sharpest at different focal settings while the rest will present varying degrees of blurriness. If chromatic aberration is strong enough, this could enable octopuses to discriminate between hues as long as there is enough spectral and spatial contrast in the scene [78,81] (but see [82]).

It is also possible that cephalopods receive polarisation cues from the environment which may be relevant for camouflage—if differences in the way objects reflect light (e.g., green algae tend to have smooth shiny laminae and so will reflect light with a higher degree of polarisation compared to red algae, which are usually smaller and more rugose) are consistent enough for different objects to have a ’polarisation fingerprint’ that octopuses can identify, or if there is a relationship between polarisation and colour, octopuses could use this information when determining the most appropriate body pattern for camouflage.

Whereas some hypotheses for mechanisms through which a colour-blind animal can achieve colour-matching have been mentioned above, how such mechanisms came about still warrants an explanation. How did a colour-blind animal develop mechanisms that allow it to match background colours? Since survival depends on effectively colour-matching in the eyes of their predators, the evolutionary drivers that shaped octopus body patterns were likely to be directly linked to their predator’s vision. Our data suggests that octopuses can colour-match only one of the tested objects (brown algae) to fool a tetrachromatic visual system, but it is likely that birds and tetrachromatic fish are not important octopus predators and so exert little selective pressure. Against monochromatic predators, such as marine mammals [53,54,55], sharks [83], moray eels [84], and other octopuses, they only need to match brightness, and so these types of predators will have exerted selective pressure for brightness-matching, a skill octopuses have all but mastered [2,17,85]. Most evolutionary pressures for colour-resemblance probably came from dichromatic and trichromatic animals, for which octopuses show adequate colour-resemblance.

## Figures and Tables

**Table 1:** Spectral sensitivities and ocular media transmission (OMT) for our four potential predator species. Spectral sensitivity shown as $\lambda_{Max}$ (wavelength of maximum absorbance) of visual pigments. OMT shown as $\lambda_{T50}$ (wavelength at which transmission falls to 50%). For oil droplets (for bird), $\lambda_{cut}$ (cut-off wavelength) and $\lambda_{mid}$ (wavelength of half-maximum absorbance) of the oil droplet are presented.

Species	Spectral Sensitivity				OMT	Oil Droplets						Source
	λ1	λ2	λ3	λ4	$\lambda_{T50}$	C$\lambda_{\mathrm{cut}}$	C$\lambda_{\mathrm{mid}}$	Y$\lambda_{\mathrm{cut}}$	Y$\lambda_{\mathrm{mid}}$	R$\lambda_{\mathrm{cut}}$	R$\lambda_{\mathrm{mid}}$	
Heller’s barracuda (*Sphyraena helleri*)	—	—	455	531								[41,46]
Blue-spotted stingray (*Neotrygon kuhlii*)	—	476	498	552	412							[47,48]
Two-spotted red snapper (*Lutjanus bohar*)	—	424	494	518	386							[49,50]
Wedge-tailed shearwater (*Puffinus pacificus*)	406	472	539	601	335	445	460	506	528	562	586	[39,51]

**Table 2:** Distance between octopus and colourful background eigenvectors.

Distance ($d_{\max}$) to Octopus Eigenvector				
	**PCs**	**Green Algae**	**Brown Algae**	**Sponge**
Non-Normalised	1 PC	0.533	0.269	0.542
	2 PCs	0.897	1	0.701
	3 PCs	0.662	0.738	0.698
Normalised	1 PC	0.932	0.999	0.574
	2 PCs	0.623	0.733	0.706
	3 PCs	0.804	0.875	0.592

**Table 3:** Fisher discriminant distance between octopus and colourful object subspaces for reflectance spectra Principal Component Analysis using 3 PCs.

Distance to Octopus Subspace			
	**Green Algae**	**Brown Algae**	**Sponge**
Non-Normalised	4.14	3.111	4.206
Normalised	6.764	4.084	7.378

**Table 4:** Distance between the cluster of octopus and background object colours (in standard deviations) for chromatic coordinates for different visual systems at surface, 10m depth, and 10m depth plus water veiling.

Distance to Octopus Subspace (σ)			
	**Green Algae**	**Brown Algae**	**Sponge**
Barracuda	4.018	3.127	5.812
Barracuda 10m	3.726	2.606	5.317
Barracuda 10m + V	3.757	2.32	3.383
Ray	5.51	3.246	6.522
Ray 10m	5.515	3.222	6.398
Ray 10m + V	4.189	2.35	4.534
Snapper	4.029	3.187	6.371
Snapper 10m	4.316	3.088	6.336
Snapper 10m + V	3.76	2.402	4.01
Bird	7.124	3.172	7.085
Bird 10m	7.55	3.779	7.040
Bird 10m + V	5.292	2.785	4.663

**Table 5:** Percentage of octopus spectra that colour-match green algae, brown algae, or sponge calculated at surface, 10m depth, and 10m depth plus water veiling using threshold values of 0.5, 1, and 1.5. Data presented as colour-match % ± binomial error interval.

Colour-Match %									
	Green Algae			Brown Algae			Sponge		
Threshold	0.5	1	1.5	0.5	1	1.5	0.5	1	1.5
Barracuda	1.563 ± 0.9	2.604 ± 1.15	2.604 ± 1.15	5.729 ± 1.68	9.896 ± 2.16	23.958 ± 3.08	0	1.042 ± 0.73	2.083 ± 1.03
Barracuda 10m	2.604 ± 1.15	2.604 ± 1.15	3.646 ± 1.35	16.667 ± 2.69	38.542 ± 3.51	69.792 ± 3.31	2.604 ± 1.15	2.604 ± 1.15	3.125 ± 1.26
Barracuda 10m + V	3.125 ± 1.26	40.104 ± 3.54	92.708 ± 1.88	58.333 ± 3.56	94.792 ± 1.6	100	48.958 ± 3.61	93.229 ± 1.81	100
Ray	0	5.208 ± 1.6	17.188 ± 2.72	6.25 ± 1.75	10.938 ± 2.25	15.104 ± 2.58	0	0	0
Ray 10m	2.083 ± 1.03	4.688 ± 1.53	10.938 ± 2.25	4.688 ± 1.53	10.938 ± 2.25	21.875 ± 2.98	0	0	0
Ray 10m + V	17.708 ± 2.75	78.125 ± 2.98	93.75 ± 1.75	43.229 ± 3.58	86.458 ± 2.47	96.354 ± 1.35	4.688 ± 1.53	57.292 ± 3.57	89.063 ± 2.25
Snapper	0	1.042 ± 0.73	2.083 ± 1.03	5.729 ± 1.68	12.5 ± 2.39	23.958 ± 3.08	0	0.521 ± 0.52	3.125 ± 1.26
Snapper 10m	0	0	1.042 ± 0.73	5.729 ± 1.68	13.542 ± 2.47	27.083 ± 3.21	0	2.6 04 ± 1.15	4.167 ± 1.44
Snapper 10m + V	3.646 ± 1.35	90.625 ± 2.1	100	89.063 ± 2.25	100	100	84.896 ± 2.58	100	100
Bird	0	0	0	1.042 ± 0.73	2.604 ± 1.15	7.292 ± 1.88	0	0	0
Bird 10m	0	0	0	0.521 ± 0.52	2.083 ± 1.03	4.167 ± 1.44	0	0	0
Bird 10m + V	0.521 ± 0.52	2.604 ± 1.15	4.688 ± 1.53	6.25 ± 1.75	34.375 ± 3.43	59.375 ± 3.54	0	0	2.083 ± 1.03

**Table 6:** Ratio of octopus spectra which effectively colour-match green algae, brown algae, or sponge, for two different visual systems. A value of 0 means that none of the octopus colours indiscernible from backgrounds to one visual system (row) are indiscernible for the other visual system (column).’–’ indicates that there are no octopus colours indiscriminable from background colours for that visual system (row).

Octopus Colourmatch Intersection Ratio													
		**Green Algae**											
		**Barracuda**			**Ray**			**Snapper**			**Bird**		
		**0.5**	**1**	**1.5**	**0.5**	**1**	**1.5**	**0.5**	**1**	**1.5**	**0.5**	**1**	**1.5**
**Barracuda**	**Surface**				0	0.8	1	0	0.4	0.8	0	0	0
	**10m**				0.8	1	1	–	0	0.286	0	0	0
	**10m + V**				1	1	1	1	948	1	0.167	0.065	0.051
**Ray**	**Surface**	–	0.4	0.152				–	0.2	0.121	–	0	0
	**10m**	1	0.556	0.333				–	0	0.095	0	0	0
	**10m + V**	0.176	0.513	0.989				0.206	0.82	1	0.029	0.033	0.05
**Snapper**	**Surface**	–	1	1	–	1	1				–	0	0
	**10m**	–	–	1	–	–	1				–	–	0
	**10m + V**	0.857	0.42	0.927	1	0.707	0.938				0.143	0.029	0.047
**Bird**	**Surface**	–	–	–	–	–	–	–	–	–			
	**10m**	–	–	–	–	–	–	–	–	–			
	**10m + V**	1	1	1	1	1	1	1	1	1			
		**Brown Algae**											
**Barracuda**	**Surface**				0.909	1	0.609	1	0.895	0.8 91	0.182	0.263	0.304
	**10m**				0.281	0.284	0.313	0.344	0.351	0.388	0.031	0.054	0.06
	**10m + V**				0.714	0.912	0.964	1	1	1	0.107	0.363	0.594
**Ray**	**Surface**	0.833	0.905	0.966				0.833	0.81	0.828	0.167	0.238	0.483
	**10m**	1	1	1				1	0.81	0.762	0.111	0.19	0.19
	**10m + V**	0.964	1	1				1	1	1	0.145	0.398	0.616
**Snapper**	**Surface**	1	0.708	0.891	0.909	0.708	0.522				0.182	0.208	0.304
	**10m**	1	1	1	0.818	0.818	0.615				0.091	0.154	0.154
	**10m + V**	0.655	0.948	1	0.485	0.865	0.964				0.07	0.344	0.594
**Bird**	**Surface**	1	1	1	1	1	1	1	1	1			
	**10m**	1	1	1	1	1	1	1	1	1			
	**10m + V**	1	1	1	1	1	1	1	1	1			
		**Sponge**											
**Barracuda**	**Surface**				–	0	0	–	0.5	1	–	0	0
	**10m**				0	0	0	0	1	1	0	0	0
	**10m + V**				0.096	0.615	0.891	1	1	1	0	0	0.021
**Ray**	**Surface**	–	–	–				–	–	–	–	–	–
	**10m**	–	–	–				0	–	–	0	–	–
	**10m + V**	1	1	1				1	1	1	0	0	0.023
**Snapper**	**Surface**	–	1	0.667	–	0	0				–	0	0
	**10m**	–	1	0.75	–	0	0				0	0	0
	**10m + V**	0.577	0.932	1	0.055	0.573	0.891				0	0	0.021
**Bird**	**Surface**	–	–	–	–	–	–	–	–	–			
	**10m**	–	–	–	–	–	–	–	–	–			
	**10m + V**	–	–	1	–	–	1	–	–	1			

## Supplementary Materials

The following supporting information can be downloaded at: https://www.mdpi.com/article/10.3390/vision6040059/s1, Figure S1: Examples of measured areas; Figure S2: Colour standard; Figure S3: Colour standard reflectance spectra; Figure 4: Error of reconstruction as a function of principal components; Figure S5: Error of reconstruction of background spectra as a function of principal components.

## Glossary

Chromaticity	The aspect of colour that remains invariant when the intensity of a light stimulus changes. Chromaticity of a colour is shown in chromaticity diagrams.
Colour	A set of signals elicited by a light stimulus in the nervous system of an animal. We quantify colour as a set of photoreceptor quantum catches (the number of absorbed photons).
Colour-Matching	Colours are matched if an animal cannot discriminate between them. We assume that colour discrimination is set by noise, which includes both fluctuations of number of absorbed quanta of light and neural noise.
Lightness	Achromatic aspect of colour relative to that of illumination. Lightness can be mediated either by a signal of a single photoreceptor spectral type or by a weighted sum of several spectral types of photoreceptors. Lightness of a reflecting surface is quantified as a ratio of a photoreceptor quantum catch (or the weighted sum) corresponding to this surface to that of the ideal white reflector.
Reflectance Spectrum	The ratio of the spectrum of light reflected from a surface to that of a surface that reflects 100% of light at all wavelengths (ideal white surface) and illuminated by the light having the same spectral composition and intensity. We often use the term `spectrum’ instead of `reflectance spectrum’ where it is clear from context that the reflectance spectrum is discussed.
Saturation	Aspect of colour that indicates how different the colour is from grey. We characterise saturation as the distance of a colour locus from an achromatic point in the Receptor Noise Limited Chromatic Diagram [36]. It is important to note that it is not known if animals perceive saturation as a distinct aspect of colour.

## References

[1] Hanlon R.T. (2007). Cephalopod Dynamic Camouflage. Curr. Biol..

[2] Hanlon R.T., Forsythe J.W., Joneschild D.E. (1999). Crypsis, conspicuousness, mimicry and polyphenism as antipredator defences of foraging octopuses on Indo-Pacific coral reefs, with a method of quantifying crypsis from video tapes. Biol. J. Linn. Soc..

[3] Hanlon R.T., Chiao C.C., Mäthger L.M., Barbosa A., Buresch K.C., Chubb C. (2009). Cephalopod dynamic camouflage: Bridging the continuum between background matching and disruptive coloration. Philos. Trans. R. Soc. B Biol. Sci..

[4] Hanlon R.T., Shashar N., Collin S.P., Marshall N.J. (2003). Aspects of the Sensory Ecology of Cephalopods. Sensory Processing in Aquatic Environments.

[5] Mäthger L.M., Hanlon R.T. (2007). Malleable skin coloration in cephalopods: Selective reflectance, transmission and absorbance of light by chromatophores and iridophores. Cell Tissue Res..

[6] Kito Y., Narita K., Seidou M., Michinomae M., Yoshihara K., Partridge J.C., Herring P.J., Rigaud J.L. (1992). A blue sensitive visual pigment based on 4-hydroxyretinal is found widely in mesopelagic cephalopods. Structures and Functions of Retinal Proteins.

[7] Matsui S., Seidou M., Horiuchi S., Uchiyama I., Kito Y. (1988). Adaptation of a Deep-sea Cephalopod to the Photic Environment. Evidence for Three Visual Pigments. J. Gen. Physiol..

[8] Michinomae M., Masuda H., Seidou M., Kito Y. (1994). Structural Basis for Wavelength Discrimination in the Banked Retina of the Firefly Squid Watasenia Scintillans. J. Exp. Biol..

[9] Seidou M., Sugahara M., Uchiyama H., Hiraki K., Hamanaka T., Michinomae M., Yoshihara K., Kito Y. (1990). On the three visual pigments in the retina of the firefly squid, Watasenia scintillans. J. Comp. Physiol. A Sens. Neural Behav. Physiol..

[10] Brown P.K., Brown P.S. (1958). Visual pigments of the octopus and cuttlefish. Nature.

[11] Messenger J.B. (1977). Evidence that Octopus is Colour Blind. J. Exp. Biol..

[12] Akkaynak D., Allen J.J., Mäthger L.M., Chiao C.C., Hanlon R.T. (2013). Quantification of cuttlefish (Sepia officinalis) camouflage: A study of color and luminance using in situ spectrometry. J. Comp. Physiol. A Neuroethol. Sens. Neural Behav. Physiol..

[13] Chiao C.C., Kenneth Wickiser J., Allen J.J., Genter B., Hanlon R.T. (2011). Hyperspectral imaging of cuttlefish camouflage indicates good color match in the eyes of fish predators. Proc. Natl. Acad. Sci. USA.

[14] Mäthger L.M., Chiao C.C., Barbosa A., Hanlon R.T. (2008). Color matching on natural substrates in cuttlefish, Sepia officinalis. J. Comp. Physiol. A Neuroethol. Sens. Neural Behav. Physiol..

[15] Froesch D., Messenger J.B. (1978). On leucophores and the chromatic unit of Octopus vulgaris. J. Zool..

[16] Hanlon R.T., Messenger J.B. (1988). Adaptive Coloration in Young Cuttlefish (*Sepia officinalis* L.): The Morphology and Development of Body Patterns and their Relation to Behaviour. Philos. Trans. R. Soc. B Biol. Sci..

[17] Messenger J.B. (1979). The eyes and skin of Octopus: Compensating for sensory deficiencies. Endeavour.

[18] Josef N., Amodio P., Fiorito G., Shashar N. (2012). Camouflaging in a complex environment-octopuses use specific features of their surroundings for background matching. PLoS ONE.

[19] Mäthger L.M., Shashar N., Hanlon R.T. (2009). Do cephalopods communicate using polarized light reflections from their skin?. J. Exp. Biol..

[20] Packard A., Sanders G.D. (1971). Body Patterns of Octopus vulgaris and Maturation of the Response to Disturbance. Anim. Behav..

[21] Anderson T.J. (1997). Habitat selection and shelter use by Octopus tetricus. Mar. Ecol. Prog. Ser..

[22] Schultz S. (2018). Heller’s Barracuda, Sphyraena Helleri. In *Fishes of Australia*. http://fishesofaustralia.net.au/home/species/2553.

[23] Bray D.J., Schultz S. (2019). Sphyraena Novaehollandiae. In *Fishes of Australia*. http://fishesofaustralia.net.au/home/species/2550.

[24] Pierce S.J., Pardo S.A., Bennett M.B. (2009). Reproduction of the blue-spotted maskray Neotrygon kuhlii (Myliobatoidei: Dasyatidae) in south-east Queensland, Australia. J. Fish Biol..

[25] Luna S.M., Froese R., Pauly D. (2019). Neotrygon Kuhlii (Müller & Henle, 1841): Blue-spotted Stingray. In *FishBass*. https://www.fishbase.in/summary/Dasyatis-kuhlii.html.

[26] OBIS (2019). Neotrygon kuhlii (Müller & Henle, 1841). Ocean Biogeographic Information System.

[27] Heithaus M. (2018). Species Fact Sheet—Rays. Shark Bay Ecosystem Research Project.

[28] Bray D.J., Red Bass, Lutjanus bohar (Forsskål 1775) (2018). In *Fishes of Australia*. http://fishesofaustralia.net.au/home/species/551.

[29] Luna S.M., Froese R., Pauly D. (2019). Lutjanus Bohar (Forsskål, 1775): Two-spot Red Snapper. In *FishBass*. https://www.fishbase.in/summary/Lutjanus-bohar.html.

[30] OBIS (2019). Lutjanus bohar (Forsskål, 1775). Ocean Biogeographic Information System.

[31] OBIS (2019). Puffinus pacificus (Gmelin, 1789). Ocean Biogeographic Information System.

[32] Szabo M., Miskelly C. (2013). Wedge-tailed Shearwater—Puffinus Pacificus (Gmelin, 1789). In *New Zealand Birds*. http://nzbirdsonline.org.nz/species/wedge-tailed-shearwater.

[33] Burger A.E. (2001). Diving Depths of Shearwaters. Auk Ornithol. Adv..

[34] Vorobyev M., Osorio D. (1998). Receptor Noise as a Determinant of Colour Thresholds. Proc. R. Soc. B.

[35] Johnsen S. (2016). How to measure color using spectrometers and calibrated photographs. J. Exp. Biol..

[36] Vorobyev M., Marshall N.J., Osorio D., Hempel de Ibarra N., Menzel R. (2001). Colourful Objects Through Animal Eyes. Color Res. Appl..

[37] Chiao C.C., Osorio D., Vorobyev M., Cronin T.W. (2000). Characterization of natural illuminants in forests and the use of digital video data to reconstruct illuminant spectra. J. Opt. Soc. Am. A Opt. Image Sci. Vis..

[38] Govardovskii V.I., Fyhrquist N., Reuter T., Kuzmin D.G., Donner K. (2000). In search of the visual pigment template. Vis. Neurosci..

[39] Hart N.S., Vorobyev M. (2005). Modelling oil droplet absorption spectra and spectral sensitivities of bird cone photoreceptors. J. Comp. Physiol. A Neuroethol. Sens. Neural Behav. Physiol..

[40] Schaefer H.M., Schaefer V., Vorobyev M. (2007). Are fruit colors adapted to consumer vision and birds equally efficient in detecting colorful signals?. Am. Nat..

[41] Marshall N.J., Vorobyev M. (2003). The design of color signals and color vision in fishes. Sensory Processing in Aquatic Environments.

[42] Johnsen S. (2012). The Optics of Life: A Biologist’s Guide to Light in Nature.

[43] Wyszecki G., Stiles W.S. (1982). Color Science: Concepts and Methods, Quantitative Data and Formulae.

[44] Kelber A., Vorobyev M., Osorio D. (2003). Animal colour vision - Behavioural tests and physiological concepts. Biol. Rev. Camb. Philos. Soc..

[45] Osorio D., Vorobyev M. (1996). Colour Vision as an Adaptation to Frugivory in Primates. Proc. R. Soc. B.

[46] Marshall N.J., Vorobyev M., Siebeck U.E., Ladich F., Collin S.P., Moller P., Kapoor B. (2006). What Does a Reef Fish See When It Sees a Reef Fish? Eating ’Nemo’ ©. Communication in Fishes.

[47] McComb D.M., Frank T.M., Hueter R.E., Kajiura S.M. (2010). Temporal Resolution and Spectral Sensitivity of the Visual System of Three Coastal Shark Species from Different Light Environments. Physiol. Biochem. Zool..

[48] Theiss S.M., Lisney T.J., Collin S.P., Hart N.S. (2007). Colour vision and visual ecology of the blue-spotted maskray, Dasyatis kuhlii Müller & Henle, 1814. J. Comp. Physiol. A Neuroethol. Sens. Neural Behav. Physiol..

[49] Lythgoe J.N., Muntz W., Partridge J.C., Shand J., Williams D.M. (1994). The ecology of the visual pigments of snappers (Lutjanidae) on the Great Barrier Reef. J. Comp. Physiol. A Sens. Neural Behav. Physiol..

[50] Siebeck U.E., Marshall N.J. (2001). Ocular media transmission of coral reef fish - Can coral reef fish see ultraviolet light?. Vis. Res..

[51] Hart N.S. (2004). Microspectrophotometry of visual pigments and oil droplets in a marine bird, the wedge-tailed shearwater Puffinus pacificus: Topographic variations in photoreceptor spectral characteristics. J. Exp. Biol..

[52] Olsson P., Lind O., Kelber A. (2018). Chromatic and achromatic vision: Parameter choice and limitations for reliable model predictions. Behav. Ecol..

[53] Dungan S.Z., Kosyakov A., Chang B.S. (2016). Spectral Tuning of Killer Whale (Orcinus orca) Rhodopsin: Evidence for Positive Selection and Functional Adaptation in a Cetacean Visual Pigment. Mol. Biol. Evol..

[54] Fasick J.I., Robinson P.R. (1998). Mechanism of Spectral Tuning in the Dolphin Visual Pigments. Biochemistry.

[55] Griebel U., König G., Schmid A. (2006). Spectral Sensitivity in Two Species of Pinnipeds (Phoca vitulina and Otaria flavescens). Mar. Mammal Sci..

[56] Fennell J.G., Talas L., Baddeley R.J., Cuthill I.C., Scott-Samuel N.E. (2019). Optimizing colour for camouflage and visibility using deep learning: The effects of the environment and the observer’s visual system. J. R. Soc. Interface.

[57] Håstad O., Victorsson J., Ödeen A. (2005). Differences in color vision make passerines less conspicuous in the eyes of their predators. Proc. Natl. Acad. Sci. USA.

[58] Pike T.W. (2018). Quantifying camouflage and conspicuousness using visual salience. Methods Ecol. Evol..

[59] Stuart-Fox D., Moussalli A. (2009). Camouflage, communication and thermoregulation: Lessons from colour changing organisms. Philos. Trans. R. Soc. B Biol. Sci..

[60] Théry M., Debut M., Gomez D., Casas J. (2005). Specific color sensitivities of prey and predator explain camouflage in different visual systems. Behav. Ecol..

[61] Wilts B.D., Michielsen K., Kuipers J., De Raedt H., Stavenga D.G. (2012). Brilliant camouflage: Photonic crystals in the diamond weevil, Entimus imperialis. Proc. R. Soc. B.

[62] Forsythe J.W., Hanlon R.T. (1997). Foraging and associated behavior by Octopus cyanea Gray, 1849 on a coral atoll, French Polynesia. J. Exp. Mar. Biol. Ecol..

[63] Messenger J.B. (1974). Reflecting elements in cephalopod skin and their importance for camouflage. J. Zool..

[64] Duarte R.C., Flores A.A., Stevens M. (2017). Camouflage through colour change: Mechanisms, adaptive value and ecological significance. Philos. Trans. R. Soc. B Biol. Sci..

[65] Wente W.H., Phillips J.B. (2003). Fixed Green and Brown Color Morphs and a Novel Color-Changing Morph of the Pacific Tree Frog Hyla regilla. Am. Nat..

[66] Langridge K.V. (2009). Cuttlefish use startle displays, but not against large predators. Anim. Behav..

[67] Langridge K.V., Broom M., Osorio D. (2007). Selective signalling by cuttlefish to predators. Curr. Biol..

[68] Norman M.D., Finn J., Tregenza T. (2001). Dynamic mimicry in an Indo-Malayan octopus. Proc. R. Soc. B.

[69] Stuart-Fox D., Whiting M.J., Moussalli A. (2006). Camouflage and colour change: Antipredator responses to bird and snake predators across multiple populations in a dwarf chameleon. Biol. J. Linn. Soc..

[70] Stuart-Fox D., Moussalli A., Whiting M.J. (2008). Predator-specific camouflage in chameleons. Biol. Lett..

[71] Marshall N.J., Messenger J.B. (1996). Colour-blind camouflage. Nature.

[72] Gomez D., Richardson C., Lengagne T., Derex M., Plenet S., Joly P., Léna J.P., Théry M. (2010). Support for a role of colour vision in mate choice in the nocturnal European treefrog (Hyla arborea). Behaviour.

[73] King R.B., Douglass J.K., Phillips J.B., Baube C.L. (1993). Scotopic spectral sensitivity of the optomotor response in the green treefrog Hyla cinerea. J. Exp. Zool..

[74] Nielsen H.I., Dyck J. (1978). Adaptation of the Tree Frog, Hyla cinerea, to Colored Backgrounds, and the Role of the Three Chromatophore Types. J. Exp. Zool..

[75] Kingston A.C.N., Kuzirian A.M., Hanlon R.T., Cronin T.W. (2015). Visual phototransduction components in cephalopod chromatophores suggest dermal photoreception. J. Exp. Biol..

[76] Mäthger L.M., Roberts S.B., Hanlon R.T. (2010). Evidence for distributed light sensing in the skin of cuttlefish, Sepia officinalis. Biol. Lett..

[77] Ramirez M.D., Oakley T.H. (2015). Eye-independent, light-activated chromatophore expansion (LACE) and expression of phototransduction genes in the skin of Octopus bimaculoides. J. Exp. Biol..

[78] Stubbs A.L., Stubbs C.W. (2016). Spectral discrimination in color blind animals via chromatic aberration and pupil shape. Proc. Natl. Acad. Sci..

[79] Land M.F., Autrum H. (1981). Optics and Vision in Invertebrates. Handbook of Sensory Physiology.

[80] Jagger W.S., Sands P.J. (1999). A wide-angle gradient index optical model of the crystalline lens and eye of the octopus. Vis. Res..

[81] Stubbs A.L., Stubbs C.W. (2016). Reply to Gagnon et al. - All color vision is more difficult in turbid water. Proc. Natl. Acad. Sci. USA.

[82] Gagnon Y.L., Osorio D.C., Wardill T.J., Marshall N.J., Chung W.S., Temple S.E. (2016). Can chromatic aberration enable color vision in natural environments?. Proc. Natl. Acad. Sci. USA.

[83] Hart N.S., Theiss S.M., Harahush B.K., Collin S.P. (2011). Microspectrophotometric evidence for cone monochromacy in sharks. Naturwissenschaften.

[84] Wang F.Y., Tang M.Y., Yan H.Y. (2011). A comparative study on the visual adaptations of four species of moray eel. Vis. Res..

[85] Hanlon R.T., Watson A.C., Barbosa A. (2010). A “Mimic Octopus” in the Atlantic: Flatfish Mimicry and Camouflage by Macrotritopus defilippi. Biol. Bull..

