# Supplementary material for: The Colours of Octopus: Using Spectral Data to Measure Octopus Camouflage
Source: Vision (Basel). 2022 Sep 22;6(4):59. doi: 10.3390/vision6040059 (PMC9590006; doi:10.3390/vision6040059)
Supplement: Supplementary file 1 [file vision-06-00059-s001.zip › vision-1834632-supplementary.pdf]

Supplementary Materials

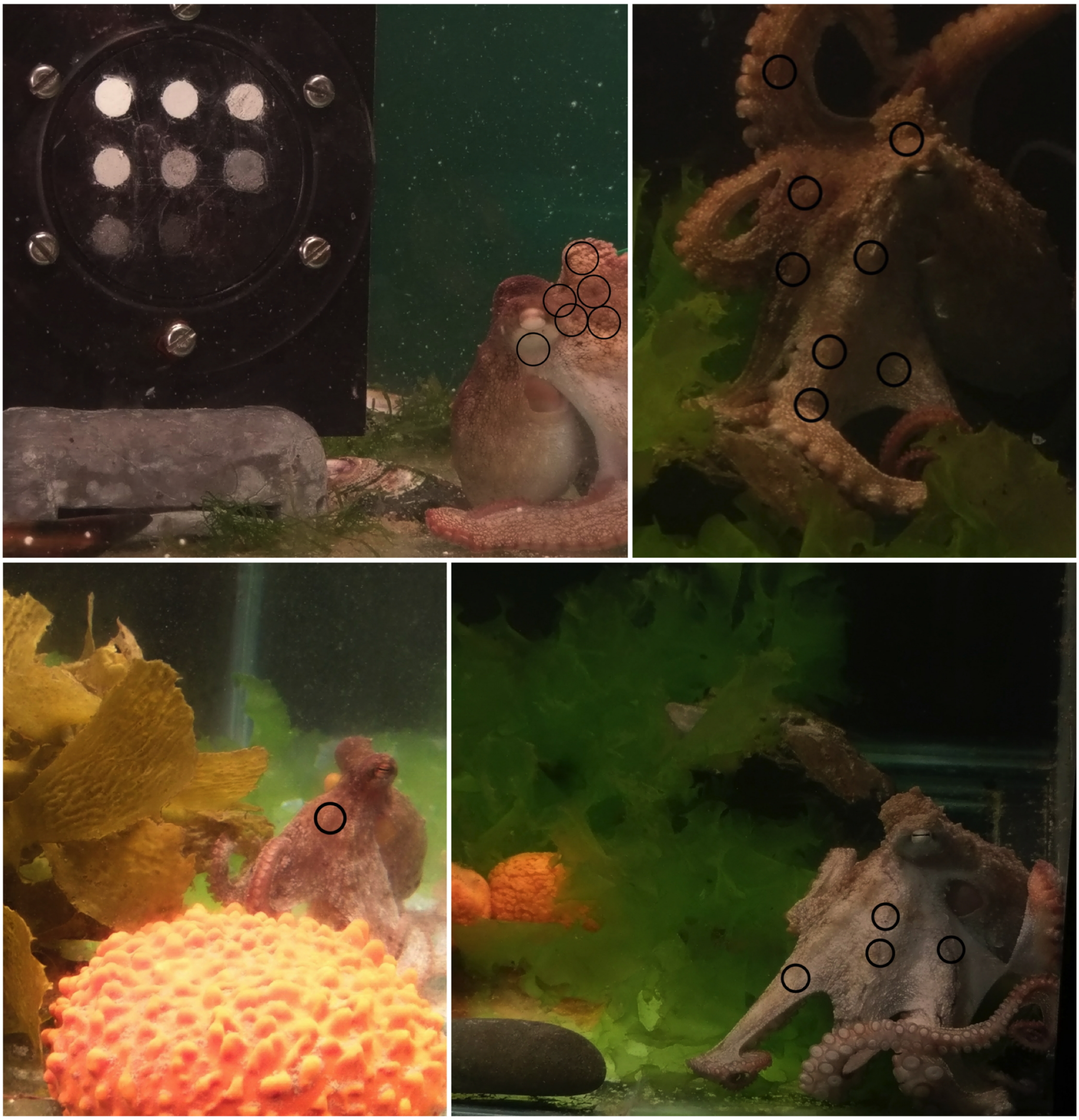

Figure S1: Examples of octopus pictures taken during spectra measurements. The black circles indicate the areas sampled with the spectroradiometer.

S1 Colour Standard

To measure the effect of the difference in the position of the white standard relative to the objects, we placed a colour standard in various locations inside the tank and measured its reflectance spectra with the spectroradiometer. The white standard was placed in the same positions as the colour standard, as well as at different locations within the tank. As with measurements of octopus and background objects, the tank was illuminated from above to minimise artefacts due to reflection from the glass walls of the tank, and standards were oriented at orthogonal or near-orthogonal angles relative to that of illumination.

The colour standard was manufactured using paint samples from Resene British Standard 5252 colour range (Resene Paints Ltd), and was laminated to allow submersion in water (see Figure S2).

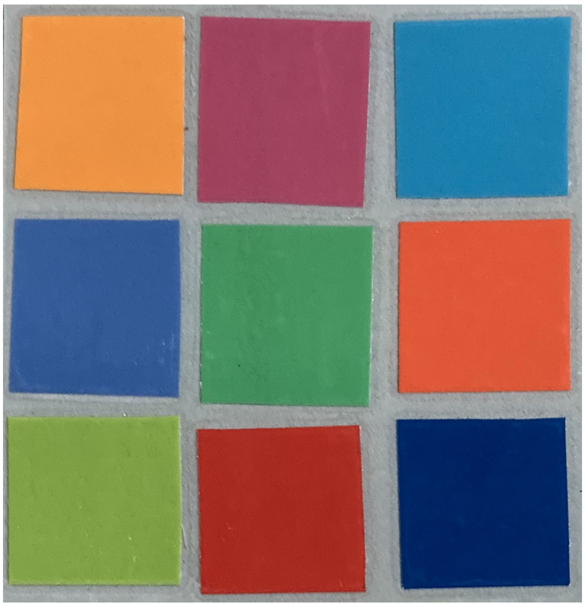

Figure S2: Colour standard manufactured with paint samples and laminated for waterproofing.

Colour standard measurements at each position were divided by the spectrum of the white standard at each location inside the tank, resulting in all possible combinations of colour standard and white standard positions. To analyse the shape of the spectra and compare the chromatic aspects between them at different positions inside the tank, we normalised them by dividing each spectrum by the area under the spectral curve (where the range of integration was 400-700nm).

Figure S3 shows that, for each colour, the shape of the reflectance spectra is very consistent. Only for 'red' & 'blue 3' (Figure S3 - bottom centre & bottom right, respectively) can it be seen that for some positions the resulting spectra are flatter. These differences can in part be due to the fact that the colour standard is laminated, as the plastic film can produce non-spectrally selective specular highlights depending on slight variations of the angle (even when viewed from a near-orthogonal angle relative to that of illumination). The overall shape of the spectra is consistent within colours, however, and each colour spectrum can be easily distinguished from all other colour spectra by their shape.

S2 Chromaticity Coordinates

The chromaticity coordinates for dichromatic, trichromatic and tetrachromatic visual systems were calculated using equations from Kelber *et al.* (2003) as:

$$X_1 = \frac{f_L - f_S}{\sqrt{{\omega_S}^2 + {\omega_L}^2}} \tag{S1a}$$

$$X_1 = \frac{f_L - f_M}{\sqrt{{\omega_M}^2 + {\omega_L}^2}}, \quad X_2 = \frac{f_S - \frac{\frac{f_M}{{\omega_M}^2} + \frac{f_L}{{\omega_L}^2}}{\frac{1}{{\omega_M}^2} + \frac{1}{{\omega_L}^2}}}{\sqrt{{\omega_S}^2 + \frac{1}{\frac{1}{{\omega_M}^2} + \frac{1}{{\omega_L}^2}}}} \tag{S1b}$$

$$X_1 = \frac{f_L - f_M}{\sqrt{{\omega_M}^2 + {\omega_L}^2}}, \quad X_2 = \frac{f_S - \frac{\frac{f_M}{{\omega_M}^2} + \frac{f_L}{{\omega_L}^2}}{\frac{1}{{\omega_M}^2} + \frac{1}{{\omega_L}^2}}}{\sqrt{{\omega_S}^2 + \frac{1}{\frac{1}{{\omega_M}^2} + \frac{1}{{\omega_L}^2}}}}, \quad X_3 = \frac{f_{VS} - \frac{\frac{f_S}{{\omega_S}^2} + \frac{f_M}{{\omega_M}^2} + \frac{f_L}{{\omega_L}^2}}{\frac{1}{{\omega_S}^2} + \frac{1}{{\omega_M}^2} + \frac{1}{{\omega_L}^2}}}{\sqrt{{\omega_{VS}}^2 + \frac{1}{\frac{1}{{\omega_S}^2} + \frac{1}{{\omega_M}^2} + \frac{1}{{\omega_L}^2}}}} \tag{S1c}$$

where ω_i denote the levels of noise in receptor mechanisms (Weber fractions).

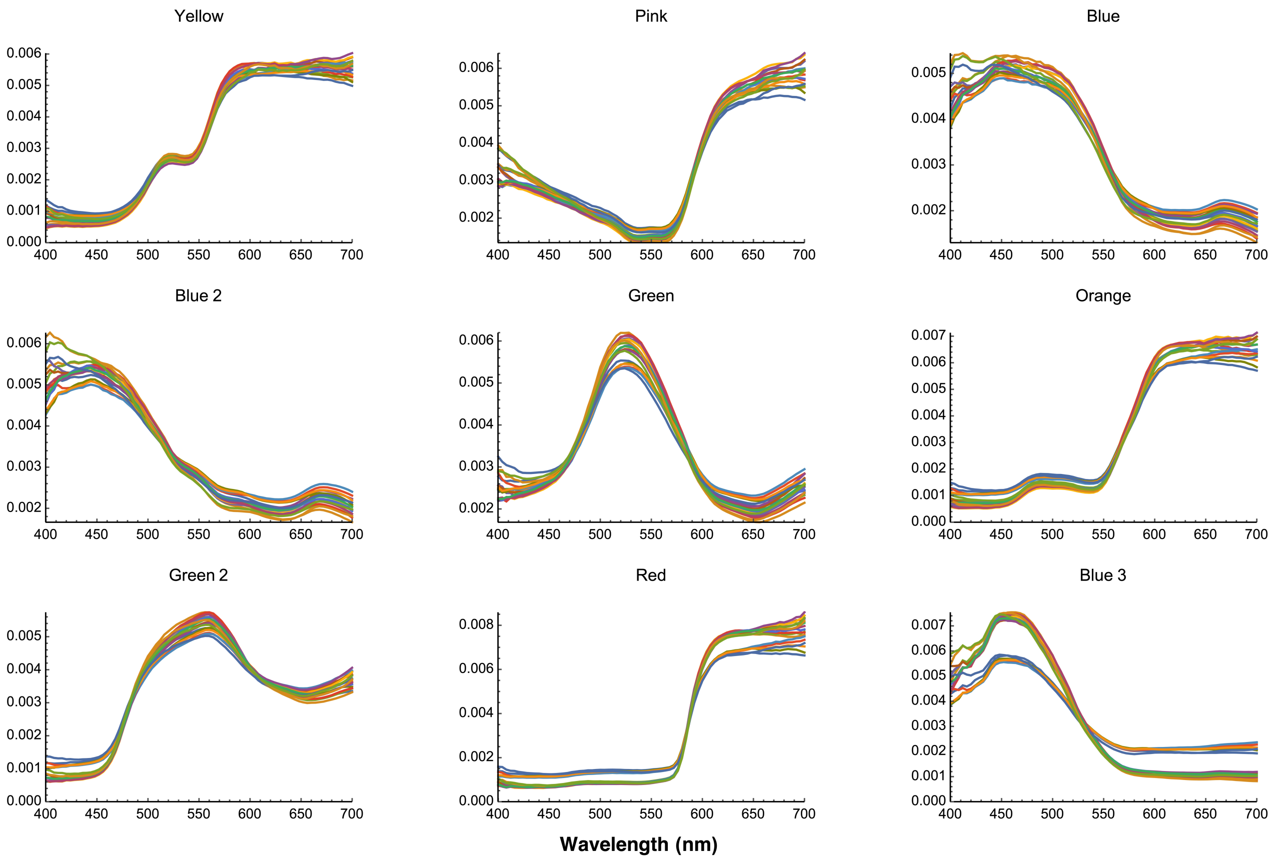

Figure S3: Comparison of colour standard reflectance spectra measured at different positions in the tank with various white standard locations. Note that the scale along the Y axis does not represent actual reflectance, as spectra have been normalised by the area under the curve.

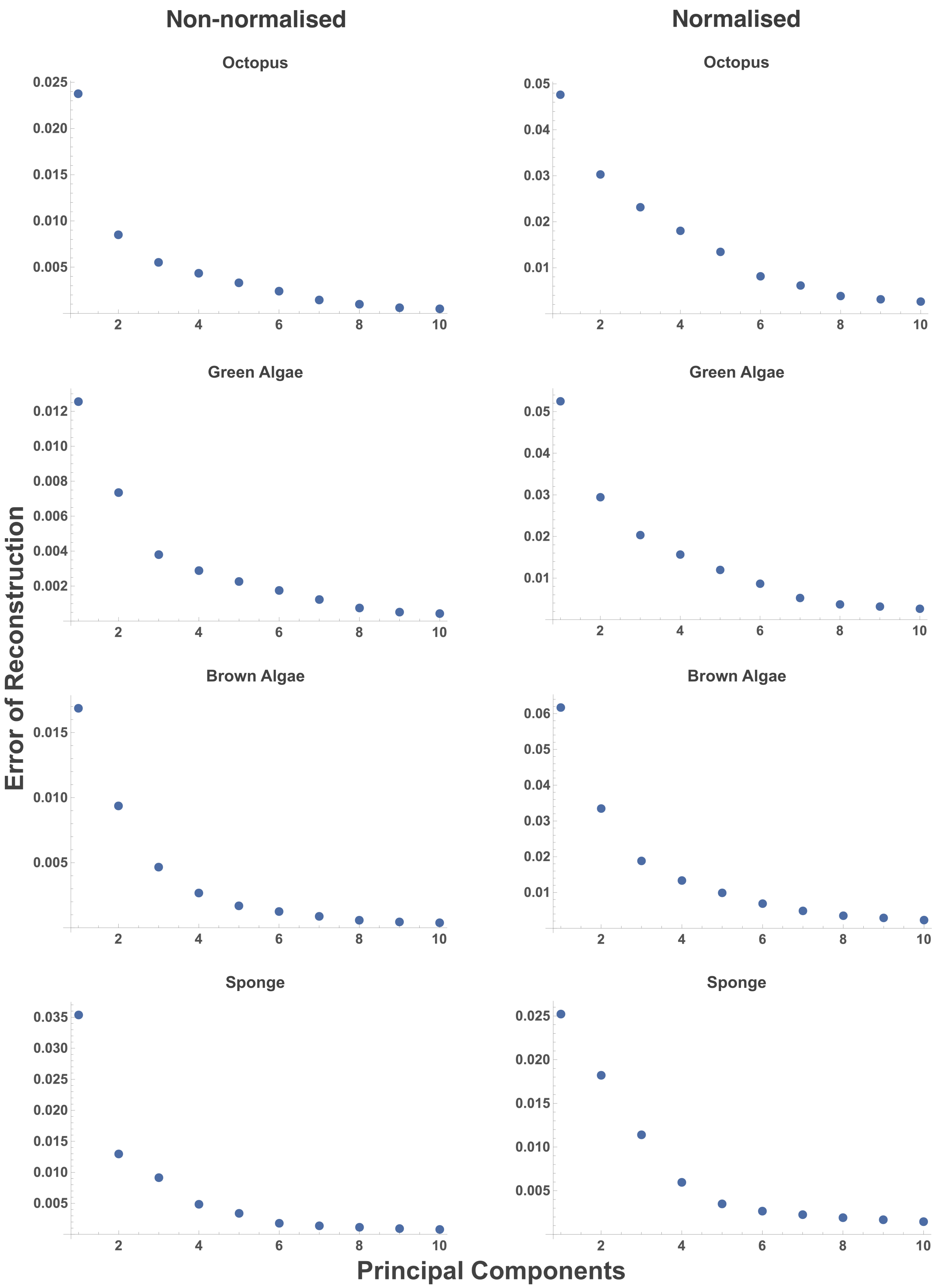

Figure S4: Error of reconstruction as a function of the number of principal components for octopus, green algae, brown algae, & sponge. For non-normalised (left) data, curves show a large decrease in error of reconstruction from one to two PCs, and a much smaller decrease after the fourth PC. Non-normalised data shows an error of 2.4%, 1.3%, 1.7%, and 3.6% for one PC of octopus, green algae, brown algae, & sponge, respectively. Normalised data increased the error of reconstruction for octopus and algae, but decreases for sponge. Error values for one PC are 4.8%, 5.3%, 6.2%, and 2.5% for octopus, green algae, brown algae, & sponge, respectively. For more than four principal components, the increase in accuracy of reconstruction is small per each additional PC for both non-normalised and normalised data. However, it is difficult to pin-point the exact number of PCs needed for reconstruction.

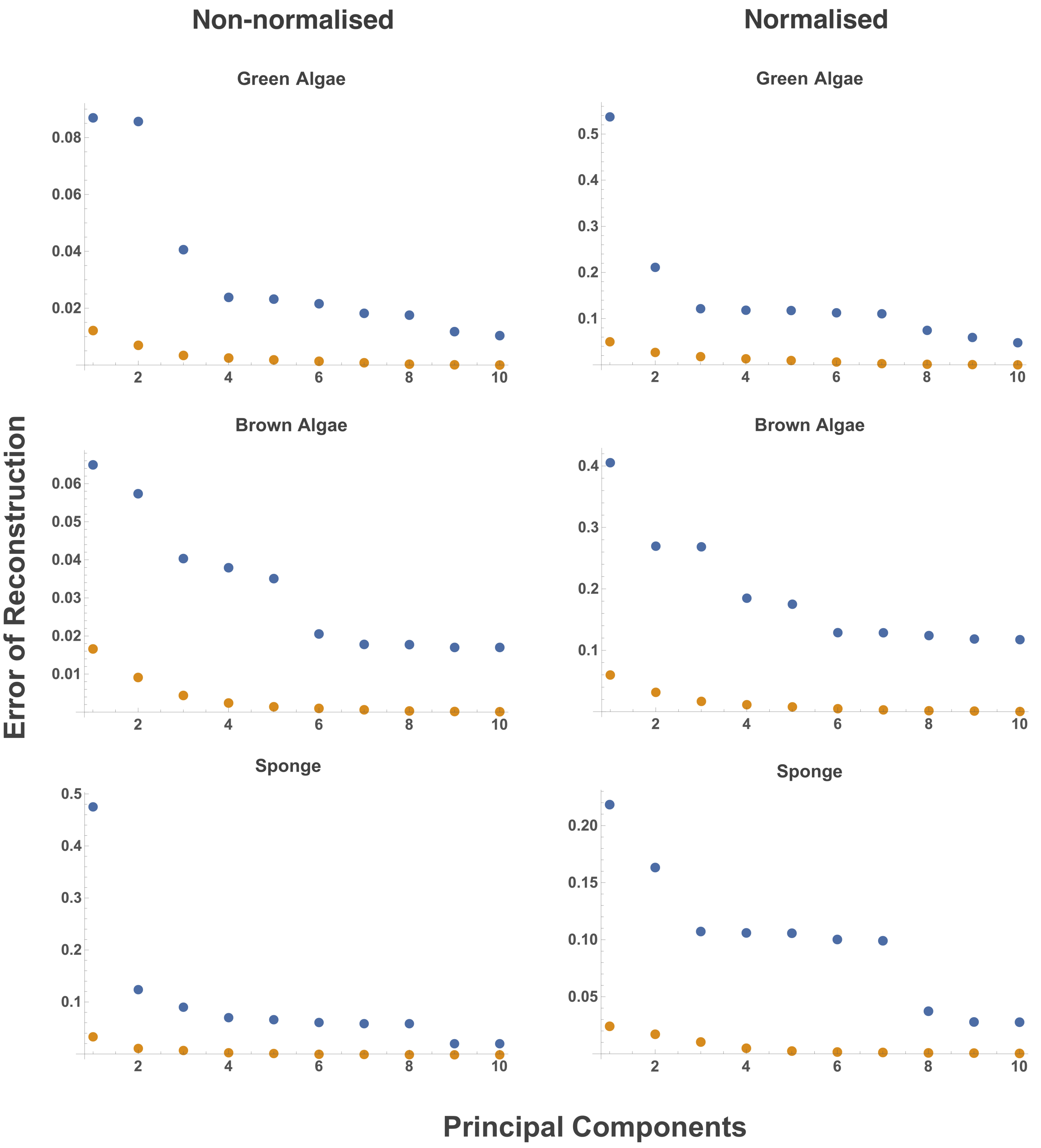

Figure S5: Error of reconstruction of backgrounds as a function of the number of principal components of octopus (blue) or of the same group (orange) for green algae, brown algae, & sponge, for both non-normalised (left) and normalised (right) spectra. It is clear that, in all cases, the error of reconstruction using PCs from the same group is substantially lower than that when using octopus PCs. An error of 8.7%, 6.5%, and 47.8% was obtained for reconstruction with one octopus PC of green algae, brown algae, & sponge, respectively, compared to 1.3%, 1.7%, and 3.6% with one own PC. Normalisation increased the error per octopus PC considerably for algae, but decreased it for sponge. For green algae, the main decrease in error of reconstruction occurs between the first three octopus PCs, thus reducing the number of octopus PCs necessary for accurate reconstruction. For brown algae and sponge, on the other hand, the decrease in error of reconstruction is much lower, thus requiring more octopus PCs for accurate reconstruction. For normalised data, the error of reconstruction using one octopus PC are 54%, 40.8%, and 22% for green algae, brown algae, & sponge, respectively, compared to 5.3%, 6.2%, and 2.5% from using one PC from the same (i.e. own) group.
